# Gut microbial diversity and functional characterization in people with alcohol use disorder: A case-control study

**DOI:** 10.1371/journal.pone.0302195

**Published:** 2024-06-12

**Authors:** Daria Piacentino, Carlotta Vizioli, Jennifer J. Barb, Silvia Grant-Beurmann, Sofia Bouhlal, Jillian T. Battista, Olivia Jennings, Mary R. Lee, Melanie L. Schwandt, Peter Walter, Wendy A. Henderson, Kun Chen, Sara Turner, Shanna Yang, Claire M. Fraser, Lisa A. Farinelli, Mehdi Farokhnia, Lorenzo Leggio

**Affiliations:** 1 Clinical Psychoneuroendocrinology and Neuropsychopharmacology Section, Translational Addiction Medicine Branch, National Institute on Drug Abuse Intramural Research Program and National Institute on Alcohol Abuse and Alcoholism Division of Intramural Clinical and Biological Research, NIH, Baltimore, MD, United States of America; 2 Interoceptive Disorders Unit, Office of the Clinical Director, National Institute of Neurological Disorders and Stroke, NIH, Bethesda, MD, United States of America; 3 Translational Biobehavioral and Health Disparities Branch, Clinical Center, NIH, Bethesda, MD, United States of America; 4 Institute for Genome Sciences and Department of Medicine, University of Maryland School of Medicine, Baltimore, MD, United States of America; 5 Office of the Clinical Director, National Institute on Alcohol Abuse and Alcoholism Division of Intramural Clinical and Biological Research, NIH, Bethesda, MD, United States of America; 6 National Institute of Diabetes and Digestive and Kidney Diseases Division of Intramural Research, NIH, Bethesda, MD, United States of America; 7 Department of Biobehavioral Health Sciences, University of Pennsylvania School of Nursing, Philadelphia, PA, United States of America; 8 Department of Statistics, University of Connecticut, Storrs, CT, United States of America; 9 Nutrition Department, Clinical Center, NIH, Bethesda, MD, United States of America; University of Illinois, UNITED STATES

## Abstract

Individuals with Alcohol Use Disorder (AUD) typically have comorbid chronic health conditions, including anxiety and depression disorders, increased sleep disruption, and poor nutrition status, along with gut microbial dysbiosis. To better understand the effects of gut dysbiosis previously shown in individuals with AUD, gut microbiome and metabolome were investigated between three cohorts. Two groups of individuals with AUD included treatment-seeking newly abstinent for at least six weeks (AB: N = 10) and non-treatment-seeking currently drinking (CD: N = 9) individuals. The third group was age, gender, and BMI-matched healthy controls (HC: N = 12). Deep phenotyping during two weeks of outpatient National Institutes of Health Clinical Center visits was performed, including clinical, psychological, medical, metabolic, dietary, and experimental assessments. Alpha and beta diversity and differential microbial taxa and metabolite abundance of the gut microbiome were examined across the three groups. Metabolites derived from the lipid super-pathway were identified to be more abundant in the AB group compared to CD and HC groups. The AB individuals appeared to be most clinically different from CD and HC individuals with respect to their gut microbiome and metabolome. These findings highlight the potential long-term effects of chronic alcohol use in individuals with AUD, even during short-term abstinence.

## Introduction

AUD is defined by the Diagnostic and Statistical Manual of Mental Disorders, Fifth Edition (DSM-5), as “a problematic pattern of alcohol use leading to clinically significant impairment or distress” [1], and both binge and heavy drinking are associated with AUD. The National Institute on Alcohol Abuse and Alcoholism (NIAAA) defines binge drinking as the consumption of ≥ 4 or 5 drinks respectively for women or men in about 2 hours, which brings the blood alcohol concentration to meet or ≥ 0.08% [2]. Furthermore, the NIAAA defines heavy drinking as the consumption of ≥ 8 or 15 drinks/week for women or men, respectively [3].

Gut microbiome and metabolome alterations have been linked to the pathophysiology of several mental health conditions, such as psychotic, depressive, and anxiety disorders [4], and, most recently, to socio-emotional impairment and alcohol craving in the context of binge drinking and AUD [5]. Chronic heavy drinking can affect all systems of the body, including the gut microbiome [6]; in fact, heavy alcohol intake has been shown to influence gut dysbiosis, gut mucosal damage, and increased gut permeability [7–10]. The latter paves the way to endotoxemia, which is the result of the translocation from the intestinal lumen to the blood circulation of microbial products, mainly lipopolysaccharide, peptidoglycans, lipoteichoic acid, and flagellin [11]. When these toxins reach the liver via the portal vein, they can induce inflammation and contribute to hepatocyte death and subsequent fibrotic response [12, 13]. Gut microbiome is affected not only by chronic heavy drinking, but also by many common diseases and prescription medications [14].

Several studies have focused on determining gut dysbiosis and metabolic dysfunction in animal models of alcohol intake and individuals with AUD. Studies in rodent models of alcohol intake found decreased gut microbial alpha- and beta-diversity (diversity within and between microbial communities, respectively), as well as differential relative abundance of phyla, such as Firmicutes and Bacteroidetes [15, 16] (the Firmicutes/Bacteroidetes ratio is typically elevated in alcohol drinking animals) [17]. Preliminary work from our group on the gut microbiome of growth hormone secretagogue receptor (GHSR) knockout vs wild-type rats after alcohol binge-like exposure showed a decrease in overall gut microbial diversity and changes in the proportion of Bacteroidales order following alcohol binge-drinking [18]. A minority of rodent studies analyzed the gut metabolome, revealing alcohol-related changes in metabolites belonging to glycerophospholipids, carboxylic acids, and fatty acyls [17, 19–21], classified as amino acids, bile acids, fatty acids, peptides, and phenols in the subclass level [21–23].

Other non-human primate studies investigated the relationship between alcohol use and the gut microbiome and metabolome [22, 23]. Two studies in *Rhesus macaques* showed an increased in inflammatory bacterial taxa, such as Firmicutes [22], and metabolomic changes primarily associated with differences in glycolysis [23]. A recent study from our group in olive baboons [24], modeling both binge and heavy drinking patterns [25, 26], found changes in the gut microbiome and metabolome, primarily loss of gut microbial alpha- and beta-diversity and enhanced energy metabolism. These changes occurred after long-term (~12 years), but not relatively short-term (~3 years) excessive drinking and were only partially affected by acute abstinence from alcohol.

Preliminary human studies point to an association between gut dysbiosis and chronic heavy drinking in individuals with AUD, typically investigated in inpatient settings [7–9]. There is evidence that alcohol misuse increases the abundance of Firmicutes and reduces Bacteroidetes [15, 27]. Fluctuations in gut luminal content of amino acids, bile acids, lipids, neurotransmitters, and markers of oxidative stress, likely reflecting alterations in the gut bacteria, have also been observed [28–30]. Individuals with greater gut dysbiosis show intestinal hyperpermeability and inflammatory response [7, 8, 10] and are also more likely to have liver damage [27]. Yet, gut dysbiosis is observed only in a subset of individuals with chronic heavy drinking [7, 8] and abstinence does not always lead to reversal of dysbiosis [7–10]. A study in individuals with AUD during detoxification found an association between gut dysbiosis, intestinal hyperpermeability, and high depression, anxiety, and alcohol craving [8], with the latter three being important predictors of relapse [31]. A study in recently abstinent inpatients with AUD found that very heavy drinkers (≥10 drinks/day) presented greater changes in the gut microbiome following abstinence vs less heavy drinkers (<10 drinks/day), yet these changes were not correlated with shifts in depression or anxiety [32].

In summary, existing human literature points to a complex relationship between AUD and gut microbial composition and function. A number of gaps exist, including the failure to account for lifestyle and environmental factors known to affect gut bacteria contributing to intra- and inter-individual heterogeneity; in addition to retrospective measures of alcohol consumption which are subject to recall bias and strictly controlled inpatient settings, failure to properly account for these factors may deter researchers from making conclusive findings on whether gut dysbiosis and metabolic alterations are due to alcohol abstinence vs factors intrinsic to inpatient care (*e*.*g*., diet, controlled environment) or other factors [14, 33]. The aim of this case-control clinical study was to analyze and compare the gut microbiome and metabolome among three groups of individuals with AUD in an outpatient setting: individuals with AUD that were both abstinent (AB) for ≥6 weeks, and non-treatment-seeking who continued drinking (CD), and healthy controls (HC) not drinking or drinking in moderation. Consistent with our previous microbiome/metabolome study in a baboon model of binge drinking [24], we hypothesized loss of gut microbial alpha- and beta-diversity and enhanced energy metabolism in the CD group, compared to the other groups. Furthermore, deep phenotyping was performed, including clinical, psychological, medical, metabolic, and dietary assessments such that other lifestyle factors could be considered to help to explain any microbiome/metabolome differences found between groups.

## Materials and methods

### Study participants

Recruitment for the current study occurred from August 28^th^ 2017 to February 25^th^ 2020 where it was terminated earlier than originally planned due to the COVID-19 pandemic. Study inclusion and exclusion criteria are listed in the **Supplementary Methods**. All participants provided written informed consent and were compensated for their time and participation. The study was approved by the NIH Institutional Review Board Approval Institution (Protocol Number: 17-DA-0093).

The three groups in the study are described as: (1) abstinent individuals (AB, N = 10), who met DSM-5 criteria for AUD [1] and recently completed at least four weeks of inpatient treatment at the NIH Clinical Center under the NIH/NIAAA treatment protocol 14-AA-0181, followed by at least two weeks of abstinence in “real life” environment upon discharge; these individuals had to continue to remain abstinent for the entire duration of the study; (2) currently drinking individuals (CD, N = 9), who met DSM-5 criteria for AUD, but were not actively seeking treatment for AUD at the time of study participation and who satisfied NIAAA criteria for heavy drinking [3] from at least four weeks prior to screening up to the day of enrollment; and healthy controls (HC, N = 12), who had no current or past diagnosis of AUD and with limiting alcohol intake to ≤ 1 or 2 drinks/day for women or men, respectively [34]. HCs were enrolled to match sex, race, mean age (±5 years), and mean BMI (±20%) of the AUD groups (ABs and CDs). In this study, drinks corresponded to U.S. standard drinking unit, containing 14 g of alcohol [35]. For a full description of the study design, see **Supplementary Methods**.

### Alcohol-related clinical assessment and psychological measures

Alcohol-related clinical characteristics and psychopathological features were assessed at screening and/or at study visits using structured clinical interviews and self-report questionnaires (for a detailed description see **Supplementary Methods**). Diagnosis of AUD and of other possible comorbid psychiatric disorders was made at screening according to the DSM-5 criteria [1] via the Structured Clinical Interview for DSM-5 (SCID-5) [36].

AUD severity was assessed at screening using the number of endorsed SCID-5 AUD criteria, the number of heavy drinking years per the Lifetime Drinking History (LDH) [37], and the total scores of the Alcohol Use Disorders Identification Test (AUDIT) [38] and the Alcohol Dependence Scale (ADS) [39]. Alcohol intake was measured via the Timeline FollowBack (TLFB) [40]: retrospectively to reflect the 90 days prior to inpatient admission for the AB group and prior to study start for the CD and HC groups. Alcohol intake was assessed at each study visit for the groups. Alcohol related variables utilized in this study included the average number of drinks per day and the number of heavy drinking days. Other alcohol related measures such as craving and withdrawal were collected using the Penn Alcohol Craving Scale (PACS) [41] and the Clinical Institute Withdrawal Assessment of Alcohol Scale, Revised (CIWA-Ar) [42]. Quality of sleep and presence of depressive and anxiety symptoms were evaluated at screening via the PSQI [43] and an abbreviated version of the Comprehensive Psychopathological Rating Scale (CPRS) [44], which encompasses the Montgomery-Åsberg Depression Rating Scale (MADRS) [45] and the Brief Scale for Anxiety (BSA) [46]. Transient mood states were assessed during the study timeframe, specifically at the first and last study visits, using the POMS anxiety and POMS depression and both were averaged between the first and last visits [47].

### Other clinical measures

#### Liver transient elastography

Transient elastography was performed using FibroScan® (Echosens, France) at the first study visit. FibroScan® is a non-invasive diagnostic ultrasound-based device used to assess liver stiffness (hardness), a measure of fibrosis, and liver fatty changes, a measure of steatosis, which are present in a variety of liver diseases [48] (see detailed description in **Supplementary Methods**). The degree of fibrosis and steatosis were quantified using a liver stiffness measurement (LSM, in kilopascals [kPa]) and a controlled attenuation parameter (CAP, in decibels/meter [dB/m]), respectively [49].

#### Medical comorbidity and medication data collection

Self-reported pre-existing medical conditions and concomitant medications were collected as part of the participants’ medical history and physical examination at the first study visit; they were then categorized as shown in **S1 Table** and based on the classification by Jackson *et al*. [14].The latter used the gut microbiome profiles of the >2,700 members of the deeply phenotyped Twins UK cohort to carry out gut microbiome association analyses with 38 common diseases (*e*.*g*., hypercholesterolemia, hypertension, type 2 diabetes, respiratory allergies, anxiety, osteoarthritis) and 51 medications (*e*.*g*., statins, proton pump inhibitors, vitamin D3, iron, calcium) that have been linked to gut dysbiosis; their findings show significant associations with gut microbial diversity measures and select taxa. Since pre-existing medical conditions and concomitant medications were reported by many participants in this study, we looked them up and cross-checked them with those listed by Jackson *et al*. to determine those linked with gut microbiota alterations (20% FDR).

#### Dietary intake assessment

Dietary intake was assessed using food records throughout study participation. At each study visit, food records were collected and reviewed by trained nutrition staff. Dietary intake reflected foods and beverages consumed prior to each study visit, starting from day after the previous visit. Gathering of dietary information corresponded to the in-clinic and at-home fecal sample collection. For further details, please see **Supplemental Methods**.

#### Gastrointestinal permeability testing

Gastrointestinal permeability, which is an index of gut mucosal integrity, was assessed using four nondigestible sugars that are excreted and measurable in the urine, with the urine sugar ratios providing a measure of permeability [50] (see full description in **Supplementary Methods**). After an overnight (≥12 hours) fast and baseline urine collection, participants drank a 100 mL solution containing sucrose (10 g/dL), lactulose (5 g/dL), mannitol (1 g/dL), and sucralose (1 g/L), and urine was collected for 5 hours. Participants were not allowed to eat or drink during these 5 hours, except for 1 L of water provided.

#### Fecal sample collection and processing

A total of 209 fecal samples were obtained from the 31 study participants which included depositions while participants were at home and also during the study visits while at the NIH Clinical Center. Fecal sample handling, processing, and analysis for microbiome and metabolome characterization have been described previously [24]. For full details of fecal sample collection and processing, see **Supplementary Methods**.

#### Gut microbiome extraction and sequencing

Gut microbiome analysis consisted of fecal DNA extraction and 16S rRNA gene sequencing, followed by data processing as described in **Supplementary Methods**.

#### Gut metabolomic profiling

Detailed methods for gut metabolite profiling are described in the **Supplementary Methods**. Following removal of metabolites with 50% or more missing values, the values were median centered, log_10_ transformed, and mean scaled prior to statistical analyses. Missing values, if any, were replaced by actual limits of detection (equal to 1/5 of the minimum positive value of each variable) [51]. The resulting metabolomics dataset comprised 1032 metabolites, all of which had known chemical identities.

### Statistics

#### Study population

We compared the study participants’ demographic, alcohol-related, psychological, and other clinical characteristics, which were non-normally distributed (all *p*’s < .05 at the Shapiro-Wilk test), using, for categorical variables, Chi-squared (*χ*^2^) test for comparisons among groups (AB *vs*. CD *vs*. HC) and between groups (AB *vs*. CD, AB *vs*. HC, CD *vs*. HC). For continuous variables, we used a Kruskal-Wallis (H) test for comparisons among groups and a Wilcoxon rank sum test for comparisons between two groups. Categorical variables were summarized using the total N sample size and the percent of the total sample, continuous variables as means and standard deviations. The JMPv16 statistical software (SAS Headquarters, Cary, NC, USA) and Prism 9.4.1 for Mac OS X (GraphPad Software, Boston, MA, USA) were used for data analysis and visualization.

#### Repeated sampling

Repeated measures for each participant’s fecal samples and dietary intake records over the six study visits were averaged to control for intra-individual variability. Repeated measures ANOVA was run to ensure that there were no statistically significant differences among study visits within participants (all *p*-values≥.05). Gut microbiome and dietary intake data presented are based on this averaging unless otherwise specified.

#### Participant characteristics

We compared the study participants’ demographic, alcohol-related, psychological, and other clinical characteristics, which were non-normally distributed (all *p*’s < .05 at the Shapiro-Wilk test), using, for categorical variables, Chi-squared (*χ*^2^) test for comparisons among groups and between groups (AB *vs*. CD, AB *vs*. HC, CD *vs*. HC). For continuous variables, a Kruskal-Wallis (H) test for comparisons among groups and a Wilcoxon rank sum test for comparisons between two groups was used. Categorical variables were summarized using the total N sample size and the percent of the total sample, continuous variables as means and standard deviations. A *p* < .05 (two-tailed) was considered statistically significant.

#### Microbiome data

Filtering and normalization, computation of microbial measures, and data visualization were conducted using MicrobiomeAnalyst v. 2.0 [52–54], JMPv16, and Prism. Data filtering parameters: minimum count = 4, 20% prevalence in samples, percentage to remove based on IQR = 10%, sample size = 10,000. The data were then normalized, reducing the ASVs from 986 to 203. Alpha diversity metrics included observed ASVs, Chao1, Shannon, and Simpson. A PCoA of Bray-Curtis distances (Bray-Curtis dissimilarity) was used to measure beta-diversity with permutational ANOVA (PERMANOVA) test for differential analyses among groups.

Three tests were used to analyze the 71 genera (from the 203 ASVs): linear discriminant analysis (LDA) effect size [55, 56], microbiome multivariable associations with linear models 2 (MaAsLin2) [57], and heat tree analysis (Wilcoxon rank sum test) [56]. Each test was used to conduct pairwise comparisons (AB *vs*. HC, AB *vs*. CD, CD *vs*. HC). LEfSe is an algorithm that first employs a Kruskal-Wallis (H) test to identify features with significant differential abundance with respect to the class of interest; then, it performs a set of pairwise tests among subclasses, using Wilcoxon rank sum test to assess the contribution of differences between groups; as a last step, it uses LDA to estimate the effect size of each differentially abundant feature and to rank the relevance of the different biological aspects [55]. A size-effect threshold of 2.0 for the logarithmic LDA score was applied for discriminative microbial features between groups. MaAsLin2 is a statistical framework using general linear models to find associations between microbial features and experimental metadata. A linear model is fit to each microbial feature that includes the primary metadata, covariate, and blocking factor variables [57]. MaAslin2 was run using age and BMI adjustment in the linear model, and covariate plots of the resulting significant genera were created. Heat tree analysis [58] displays statistics associated with taxa (relative abundances) in a tree format. It leverages the hierarchical nature of the data to depict taxonomic differences quantitatively and statistically (Wilcoxon rank sum test) between microbial communities.

Genera found to be statistically significant at any of the three tests are reported. All testing procedures selected genera at *p* < .05. Statistically significant genera were assessed for multiple comparisons and none passed the adjustment, thus the genera *p*-values reported are unadjusted.

#### Metabolomic data

A total of 1032 identified metabolites were used to investigate metabolomic profiles among and between groups using both a Kruskal-Wallis [7] [H] test for and post-hoc Wilcoxon rank sum test for pairwise comparisons. Any metabolite with missing values in ≥ 50% of the samples was excluded from the analysis. Data were normalized by median centering, and mean scaling and were further transformed using a logarithm base 10 transformation. The final dataset submitted for analysis after filtering was 946 metabolites. Multiple comparisons were controlled for using a Benjamini-Hochberg (FDR) correction [59] and those passing a corrected *p* < .05 (KW test) were considered significant. A multivariate unsupervised principal component analysis (PCA) and supervised analyses (partial least squared discriminant analysis [PLS-DA]) were carried out using MetaboAnalyst v. 5.0 [60]. Variable importance in projection (VIP) measures were extracted in PLS-DA and calculated as a weighted sum of the squared correlations between the PLS-DA components and the original variable.

#### Gut microbiome and metabolome data correlation

To integrate the microbiome and the metabolome datasets, a correlation analysis between differential gut taxa and differential metabolites was performed using Spearman’s correlation. Relative taxa abundances and normalized Log10 transformed metabolites were used in the Spearman correlation analysis. A significant association was reported if the association was found to have an absolute Spearman ρ ≥0.50 and *p* < .05 (unadjusted). In addition, a Spearman’s correlation analysis was conducted between all taxa/metabolites within each group in R (Hmisc package v. 5.1–0). Only bacterial genera and fecal metabolites that had more than 50% non-zero values across participants were included in the analysis. Multiple comparisons were corrected using Bonferroni’s correction [61]. A microbiome-metabolite correlation was included in the results if Bonferroni corrected *p* < .05 and an absolute Spearman’s ρ of ≥0.85 was found. Significant microbiome-metabolite correlations within each group are presented using circos plots (R package circlize v. 0.4.16) in the **Supplemental Material**.

#### Study approval

The study was approved by the NIH Institutional Review Board. All participants provided informed consent and were compensated for their time and participation.

## Results

### Study population demographic and laboratory characteristics

**Fig 1:**
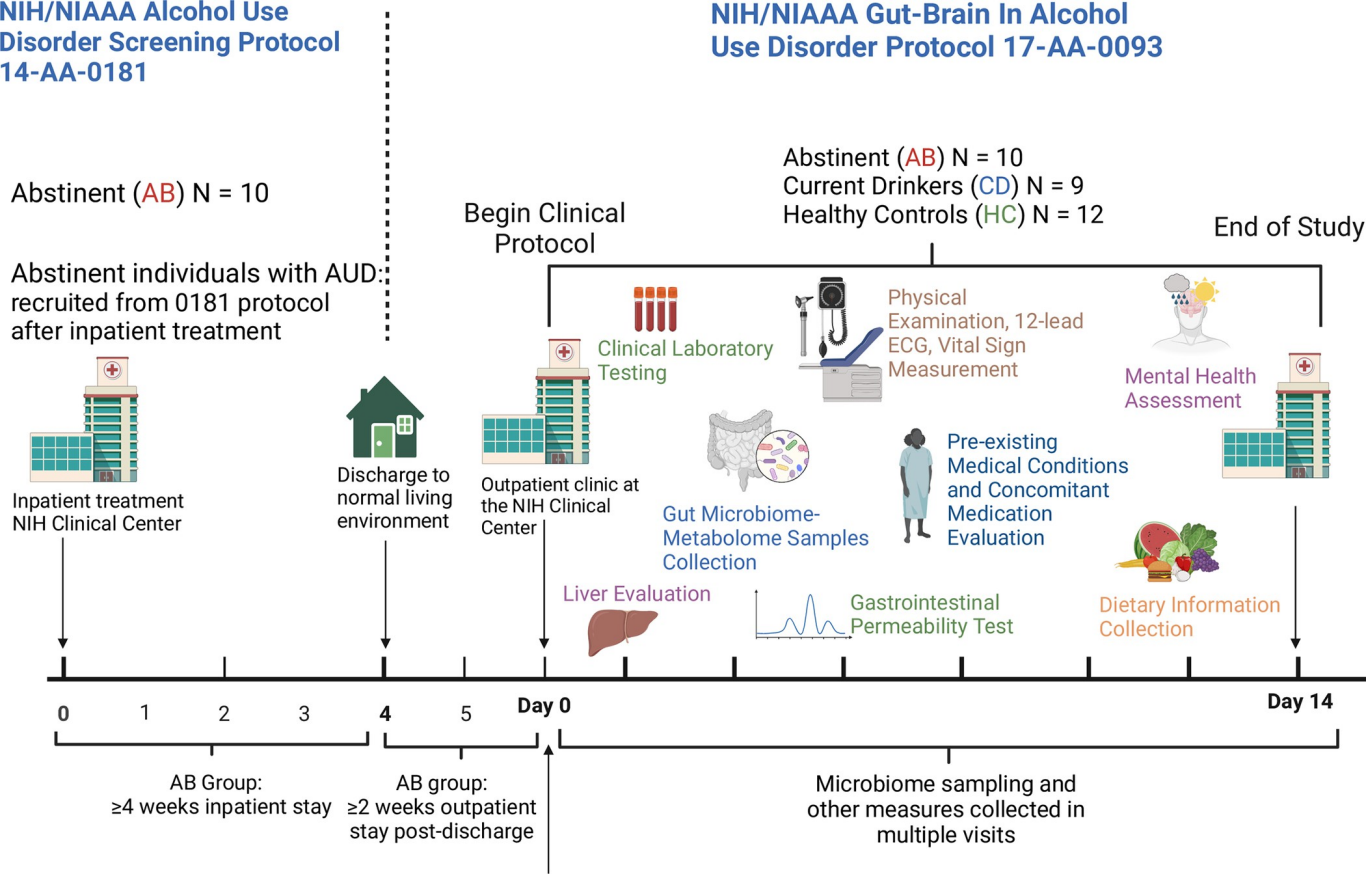
Schematic representation of the study design. Abstinent individuals with AUD (AB) were enrolled in the study after ≥4 weeks of inpatient treatment (NIH/NIAAA treatment protocol 14-AA-0181), followed by ≥2 weeks of “real life” (living their normal life). Non-treatment-seeking, currently drinking individuals with AUD (CD) and matched healthy controls (HC) were also enrolled. Fecal samples from the study participants were collected and processed for gut microbiome and metabolome analysis. Physical examination, 12-lead ECG, vital sign measurements, and laboratory tests were performed. Information on physical and mental health (including information on medical conditions and medications) and dietary intake was gathered and analyzed. Transient liver elastography and gastrointestinal permeability testing were carried out.

Enrollment and data collection for this study included six outpatient study visits (over 14-days ranging between 6–20 days in total) to the NIH Clinical Center (**Fig 1**).

Main demographic and laboratory characteristics of the groups are presented in **Table 1**. Overall, no significant differences in sex, race, ethnicity, age, and BMI were observed among groups. As expected, ABs and CDs had a higher proportion of smokers than HCs, who were all non-smokers (*p* = .007). Average liver function tests during the study were within normal range and did not differ among groups. Of note, we found higher values of AST (AB: 55.8±49.9, CD:21.8±6.00, HC:22.3±7.03, *p* = .023) and GGT (AB:134±175, CD:30.8±12.3, HC:22.0±12.8, *p* = .0032) in the AB group collected under 14-AA-0181 screening protocol, *i*.*e*., before the inpatient treatment at the NIH Clinical Center. The inflammatory biomarker c-reactive protein (CRP) and other parameters, including blood cell counts, coagulation factors, lipids, thyroid hormones, and urinalysis were all within normal range and did not differ among groups. At the FibroScan®, no participant had an exclusionary LSM score ≥17.6 kPa. FibroScan® data showed no difference among groups in LSM and CAP.

**Table 1:** Main characteristics of study population.

Variable		Abstinent (AB, N = 10)	Current Drinkers (CD, N = 9)	Healthy Controls (HC, N = 12)	Test Statistic	*P*-value
***Demographic***						
Sex, N (%)	Female	2 (20.0)	2 (22.2)	4 (33.3)	*χ*^2^ = 0.6	.744
	Male	8 (80.0)	7 (78.8)	8 (66.7)		
Race, N (%)	White/Caucasian	6 (60.0)	7 (77.8)	7 (58.3)	*χ*^2^ = 3.1	.546
	Black/African American	3 (3.0)	2 (22.2)	5 (41.7)		
	Asian	1 (1.0)	0	0		
Ethnicity, N (%)	Hispanic/Latino	0	0	1 (8.4)	*χ*^2^ = 1.6	.441
	Non-Hispanic/Latino	10 (100.0)	9 (100.0)	11 (91.6)		
Age, years, mean (SD)		45.9 (11.4)	45.0 (12.6)	48.8 (12.0)	H = 0.5	.766
BMI, kg/m^2^, mean (SD)		26.7 (5.1)	25.7 (4.0)	28.8 (4.9)	H = 2.4	.299
Smoking status, N (%)	Smoker	6 (60)	4 (44.4)	0	*χ*^2^ = 9.8	**.007**
	Non-Smoker	4 (40)	5 (55.5)	12 (100.0)		
***Laboratory*** mean (SD)						
ALT (U/L)		22.7 (13)	22.9 (11.4)	22.7 (10.7)	H = 0.1	.973
AST (U/L)		24.9 (9.6)	22.3 (6.3)	22.1 (5.4)	H = 0.3	.861
ALP (U/L)		83.2 (46.9)	64.6 (12.9)	70.2 (28.1)	H = 0.4	.801
GG (U/L)		47.6 (72.3)	36.6 (18.1)	22 (13.4)	H = 3.8	.149
Total bilirubin (mg/dL)		0.6 (0.3)	0.5 (0.2)	0.5 (0.3)	H = 0.4	.824
CRP (mg/dL)		1.4 (0.8)	2.8 (1.6)	2.1 (2.8)	H = 0.8	.654
***Alcohol-related*** mean (SD)						
Number of DSM-5 AUD criteria		9 (2)	5.5.	0	H = 26.8	**< .001**
AUDIT		28 (7.7)	16 (5.6)	2 (1.6)	H = 24.7	**< .001**
ADS		22 (9.2)	10 (6.1)	0	H = 25.2	**< .001**
PACS		7 (5)	17.5	0.5	H = 20.4	**< .001**
LDH heavy drinking years		16.4 (9.4)	12.1 (13.8)	0	H = 15.2	**< .001**
CIWA-Ar		0.9 (1.1)	0.33 (0.7)	0 (0.0)	H = 2.3	.294
90-day TLFB average drinks/day^a^		13.6 (8.0)	3.7 (1.2)	0.3 (0.3)	H = 25.7	**< .001**
90-day TLFB heavy drinking days^a^		72.6 (23.6)	66.0 (20.8)	13.6 (14.3)	H = 20.4	**< .001**
In-study average drinks/day^b^		0 (0)	3.6 (0.7)	1.4 (0.6)	H = 9.3	**.007**
***Psychological mean*** (SD)						
PSQI		12 (3.5)	5 (4.0)	2.5 (4.0)	H = 16.2	**< .001**
MADRS^c^		15 (7.8)	0.38 (1.1)	1.3 (1.7)	H = 19.1	**< .001**
BSA^c^		11 (6.6)	0.11 (0.3)	1.2 (1.7)	H = 21.1	**< .001**
POMS depression^d^		5.3 (6.6)	3.9 (4.5)	1.2 (1.7)	H = 7.5	**.023**
POMS anxiety^d^		4.3 (3.0)	3.4 (2.6)	2.4 (1.8)	H = 2.5	.292
FibroScan® mean (SD)						
LSM^e^		4.4 (1.5)	4.5 (1.2)	4.0 (0.9)	H = 0.9	.624
CAP^e^		229.5 (79.7)	220.1 (80.4)	244.3 (69.7)	H = 0.2	.916
Medical conditions and medications mean (SD)						
Number of conditions		4.0 (1.8)	1.3 (1.2)	1.6 (1.3)	H = 11.1	**.004**
Number of medications		2.2 (1.6)	0.7 (1.4)	0.7 (1.1)	H = 7.2	**.028**

Significant *p*-values (Benjamini-Hochberg corrected) in bold. Abbreviations: ADS = Alcohol Dependence Scale; AUDIT = Alcohol Use Disorders Identification Test; BMI = body mass index; BSA = Brief Scale for Anxiety; CAP = Controlled Attenuation Parameter; CIWA-Ar = Clinical Institute Withdrawal Assessment of Alcohol Scale, Revised; LDH = Lifetime Drinking History; LSM = Liver Stiffness Measurement; MADRS = Montgomery-Åsberg Depression Rating Scale; PACS = Penn Alcohol Craving Scale; POMS = Profile of Mood States; PSQI = Pittsburgh Sleep Quality Index; SD = Standard Deviation; TLFB = Timeline FollowBack.

^a^Prior 90-day TLFB reflects 90 days prior to inpatient admission for ABs and 90 days prior to study start for CDs and HCs.

^b^In-study TLFB reflects days of study participation for the groups.

^c^MADRS and BSA were administered at screening.

^d^POMS depression and POMS anxiety scores are mean scores of the first and last study visits.

^e^LSM and CAP scores correspond to the median values of 10 valid measurements. Counts and percentages (%) are shown for categorical variables; means/standard deviations (SDs) are shown for continuous variables. Chi-squared (*χ*^2^) test was used for categorical variables and Kruskal-Wallis (H) test was used for continuous variables. Normal range of alanine transferase (ALT) is 23–69 U/L, of aspartate transferase (AST) is 19–62 U/L, of alkaline phosphatase (ALP) is 0–555 U/L, of gamma-glutamyl transferase (GGT) is 5–40 U/L of total bilirubin is 0.1–1.2 mg/dL of c-reactive protein (CRP) is 0.3–1.0 mg/dL.

### Alcohol-related clinical and psychological characteristics

**Fig 2:**
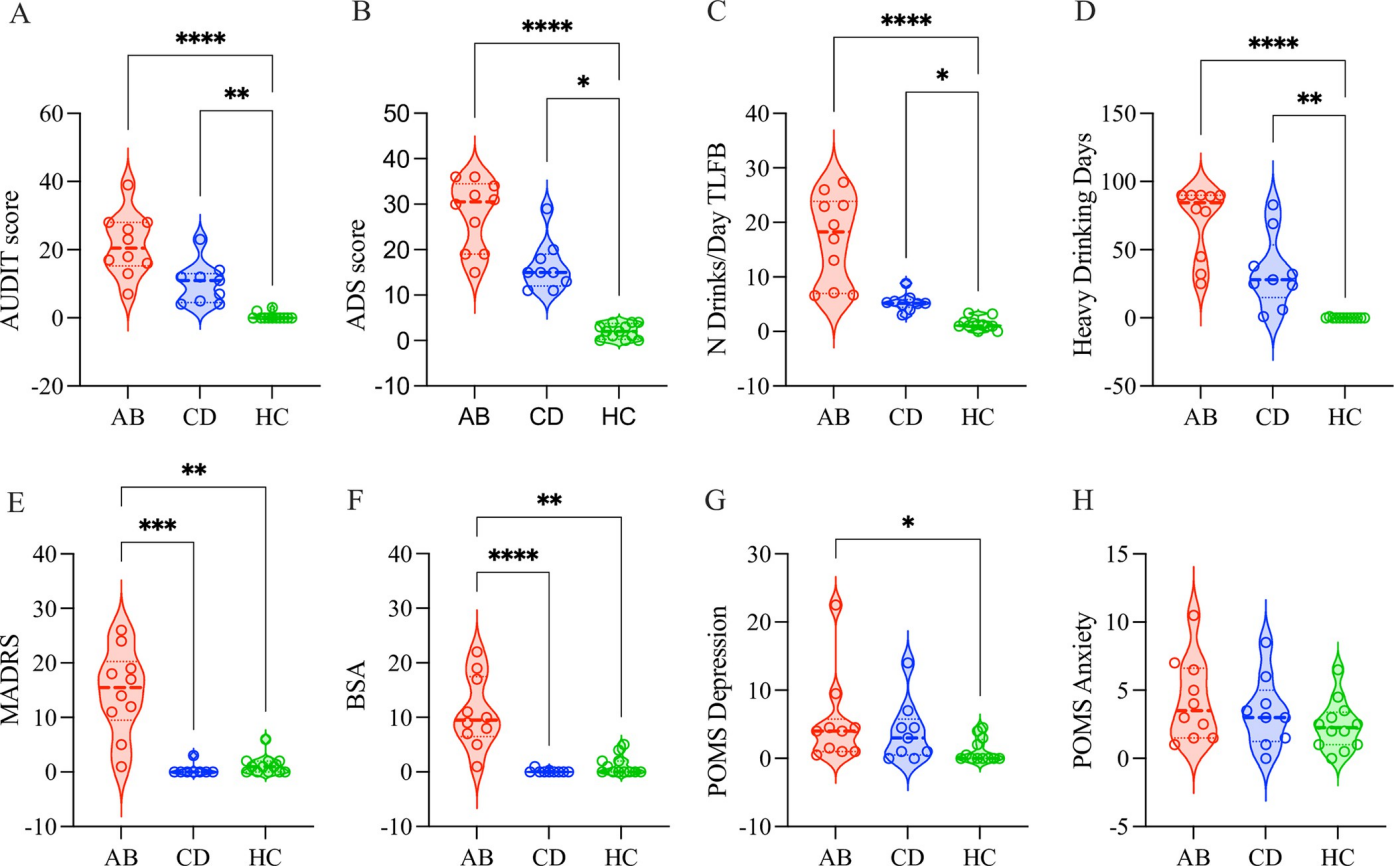
AUD severity and mental health status. (A) AUDIT scores were significantly different among groups (p < .001) and between groups (AB *vs*. HC, p < .001; CD *vs*. HC, p = .012). (B) ADS scores were significantly different among (p < .001) and between groups (AB *vs*. HC, p = .001; CD *vs*. HC, p < .009). (C) Prior 90-day TLFB average drinks per day were significantly different among (p < .001) and between groups (AB *vs*. HC, p < .001; CD *vs*. HC, p = .019). (D) Prior 90-day TLFB heavy drinking days were significantly different among (p < .001) and between groups (AB *vs*. HC, p < .001; CD *vs*. HC p = .007). (E) MADRS scores were significantly different among (p < .001) and between groups (AB *vs*. HC, p < .001; AB *vs*. CD p = .003). (F) BSA scores were significantly different among (p < .001) and between groups (AB *vs*. HC, p < .001; AB *vs*. CD, p < .001). (G) POMS depression scores were significantly different among (p < .023) and between groups (AB *vs*. HC, p < .001). (H) POMS anxiety scores did not differ significantly among and between groups.

Average AUDIT scores were different among groups (*p* < .001) and between groups (AB *vs*. HC, *p* < .001; CD *vs*. HC, *p* = .012) (**Fig 2A**). ADS scores were different among (*p* < .001) and between groups (AB *vs*. HC, *p* = .001; CD *vs*. HC, *p* < .009) (**Fig 2B**). Per prior 90-day TLFB, ABs and CDs reported more average drinks per day than HCs (*p* < .001 and *p* = .019, respectively, **Fig 2C**), as well as more heavy drinking days than HCs (*p* < .001 and *p* < .007, respectively, **Fig 2D**). Average drinks per day and heavy drinking days between ABs and CDs were not significant (*p* = .074 and *p* = .285, respectively), however in both cases, the averages were higher for ABs. Depression (MADRS), anxiety (BSA) and sleep quality (PSQI) were evaluated across all individuals at screening, and all were different among groups (*p*’s < .001) (**Fig 2E and 2F**); ABs had higher depression than CDs and HCs (*p* < .001 and *p* = .003, respectively) and higher anxiety than CDs and HCs (*p <* .001 and *p* < .001, respectively). Interestingly, CDs and HCs did not differ in depression and anxiety. POMS depression scores were different among groups (*p* < .023) and the only significant difference was between ABs and HCs (*p* < .001) (**Fig 2G**). POMS anxiety scores were higher but not significant among groups (**Fig 2H**).

### Gut microbiome across groups

**Fig 3:**
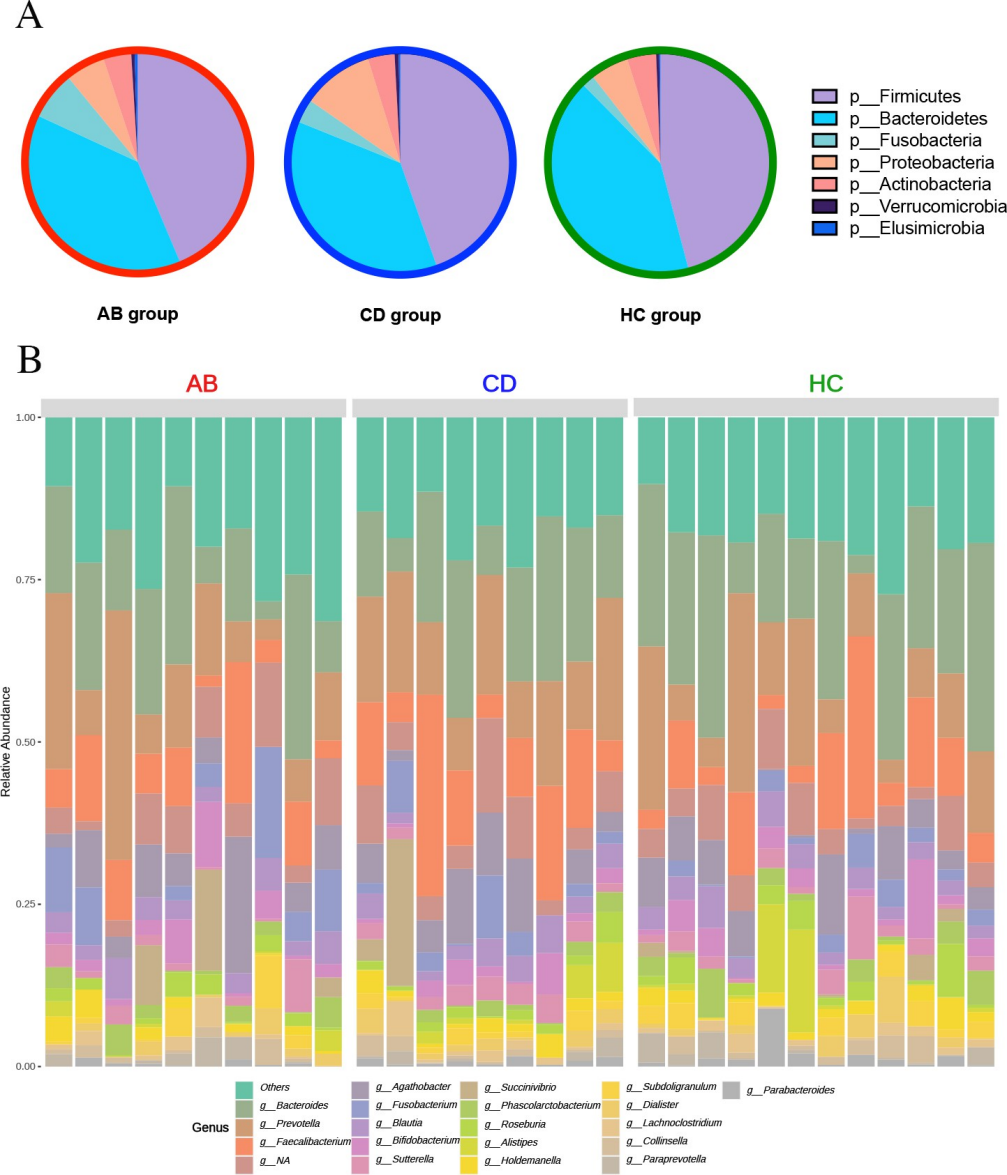
Gut microbiome of study participants. (**A**) Phylum-pie chart across entire study population. Legend shows top seven most abundant phyla. (**B**) Relative abundance bar chart at genus level across all participants. Legend shows the top 20 most abundant genera.

The overall distribution of the phyla across the groups showed that Firmicutes was most abundant with Bacteroides being second most dominant. Actinobacteria represented 6.19%, 2.56% and 3.34% in ABs, CDs, and HCs, respectively, and Proteobacteria represented 5.74%, 10.77%, and 8.14%, respectively (**Fig 3A**). At the genus level, *Bacteroides* was the most abundant genus in all groups, with a relative abundance of 21.30% in ABs, 17.87% in CDs, and 23.61% in HCs. *Faecalibacterium* and *Agathobacter* were second and third most dominant genera. (**Fig 3B**).

### Gut microbial alpha- and beta-diversity

**Fig 4:**
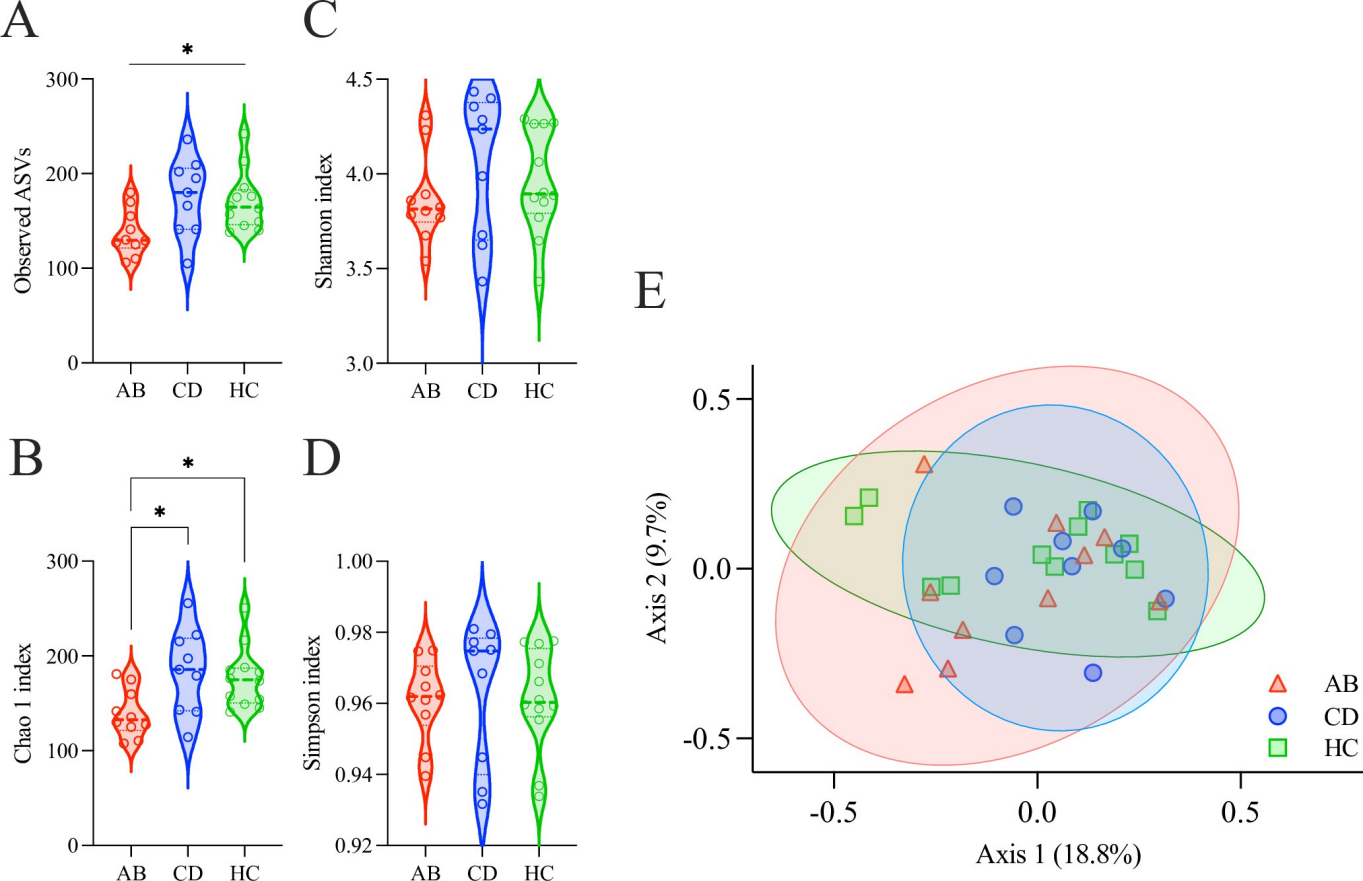
Alpha- and beta-diversity of the gut microbiome. (**A**) Alpha-diversity, calculated with Observed ASVs richness, differed significantly among ABs, CDs, and HCs (p = .026). (**B**) Alpha-diversity, calculated with Chao1, differed significantly among groups (p = .012). (**C**) Shannon diversity index did not differ significantly among groups (p = .376). (**D**) Simpson diversity index did not differ significantly among groups (p = .626). (**E**) Principal coordinates analysis (PCoA) plot comparing gut microbial beta-diversity (Bray-Curtis dissimilarity) among ABs, CDs and HCs, exhibited no separation among groups.

In total, 986 ASVs (618,267 reads) were identified across the 31 study participants (19,944 average reads/participant). Alpha diversity measures Chao1, Shannon, Simpson, and observed ASVs indexes were calculated on the full set of 986 ASVs. Average alpha diversity measures showed only species richness (Observed ASVs and Chao1) exhibiting significant differences among groups (**Fig 4A–4D and Table 2**).

**Table 2:** Alpha diversity measures.

Alpha-Diversity Indexes	Abstinent (AB, N = 10)	Current Drinkers (CD, N = 9)	Healthy Controls (HC, N = 12)	Kruskal-Wallis	*P-*value
Observed ASVs	137 (24.32)	175 (40.81)	171 (30.95)	H = 7.263	**.026**
Chao1	139 (25.22)	184 (44.88)	177 (31.90)	H = 8.835	**.012**
Shannon	3.87 (0.24)	4.05 (0.38)	3.96 (0.28)	H = 1.954	.376
Simpson	0.96 (0.01)	0.963 (0.02)	0.961 (0.01)	H = 0.936	.626

Means and standard deviations (SDs) are shown for each alpha-diversity index. Kruskal-Wallis (H) test was used to compare the median of each index among the three study groups, *i*.*e*., abstinent (AB) current drinkers (CD), and healthy controls (HC). *p* < .05 shown in bold. Abbreviations: ASV = Amplicon Sequence Variant; SD = Standard Deviation.

There were no significant beta diversity differences observed among groups using Principal Coordinate of Analysis (PCoA) (**Fig 4E**, F-value = 0.911; *R*-squared = 0.061; *p* = .619). Alpha diversity measures (Observed, Chao1, Simpson, and Shannon) were correlated with alcohol severity scores (AUDIT and ADS) within each group and no significant associations were observed.

### Group wise genus-level differential abundance

The 986 ASVs were filtered, removing low abundance and low variance features, resulting in 203 ASVs that classified to 71 genera. Differential abundance was assessed between groups, *i*.*e*., AB *vs*. CD, AB *vs*. HC, and CD *vs*. HC using three testing procedures: LEfSe, MaAsLin2, and heat tree analysis (Wilcoxon rank sum test).

Twelve taxa were found to be significantly different between groups in at least one test (no adjustment). Taxa relative abundances and fold changes of the pairwise comparisons are shown in **Table 3.**

**Table 3:** Log_2_ fold change of 12 genera found to be significantly different between groups.

	Relative Abundance (%) Mean (SD)			Log_2_ Fold Change		
Taxa	AB	CD	HC	AB *vs*.CD	AB *vs*. HC	CD *vs*. HC
*Akkermansia*	0.936	1.084	1.297	-3.8 ⱡ**↓**	-2.19**↓**	-1.61**↓**
***Candidatus Stoquefichus***	0.169	0.001	0.049	**3.02** * **↑**	1.34 **↑**	1.69**↑**
*Dorea*	0.802	2.150	0.897	-1.35^¥^**↓**	0.265**↑**	-1.61^¥^**↓**
***Eisenbergiella***	0.147	0.012	0.027	**1.79** * **↑**	1.27**↑**	0.515**↑**
***Fusicatenibacter***	1.194	1.971	2.499	-3.77^¥^**↓**	**-3.92** * **↓**	0.152**↑**
***Lachnospira***	0.560	0.719	1.658	-1.44**↓**	**-1.95** * **↓**	0.51 ⱡ**↑**
*Lachnospiraceae_FCS020_group*	0.003	0.016	0.059	-0.47**↓**	-1.27**↓**	0.798**↑**
***Lachnospiraceae_UCG_001***	0.000	0.065	0.093	-0.613**↓**	**-1.32** * **↓**	0.704**↑**
***Roseburia***	0.841	1.386	3.961	-1.66**↓**	**-2.29** * **↓**	0.632**↑**
*Ruminococcaceae_UCG_014*	0.100	0.362	1.198	-2.09**↓**	-2.48^¥^**↓**	0.39**↑**
***Streptococcus***	0.614	0.140	0.129	**2.44** * **↑**	**2.63** * **↑**	-0.193**↓**
*Victivallis*	0.008	0.199	0.108	-4.39**↓**	-1.66**↓**	-2.73 **↓**

*p < .05 on all 3 tests

ⱡ *p* < .05 only on 2 tests

^¥^ *p* < .05 only on 1 test. ↑ and ↓ indicates minuend direction of change.

**Fig 5:**
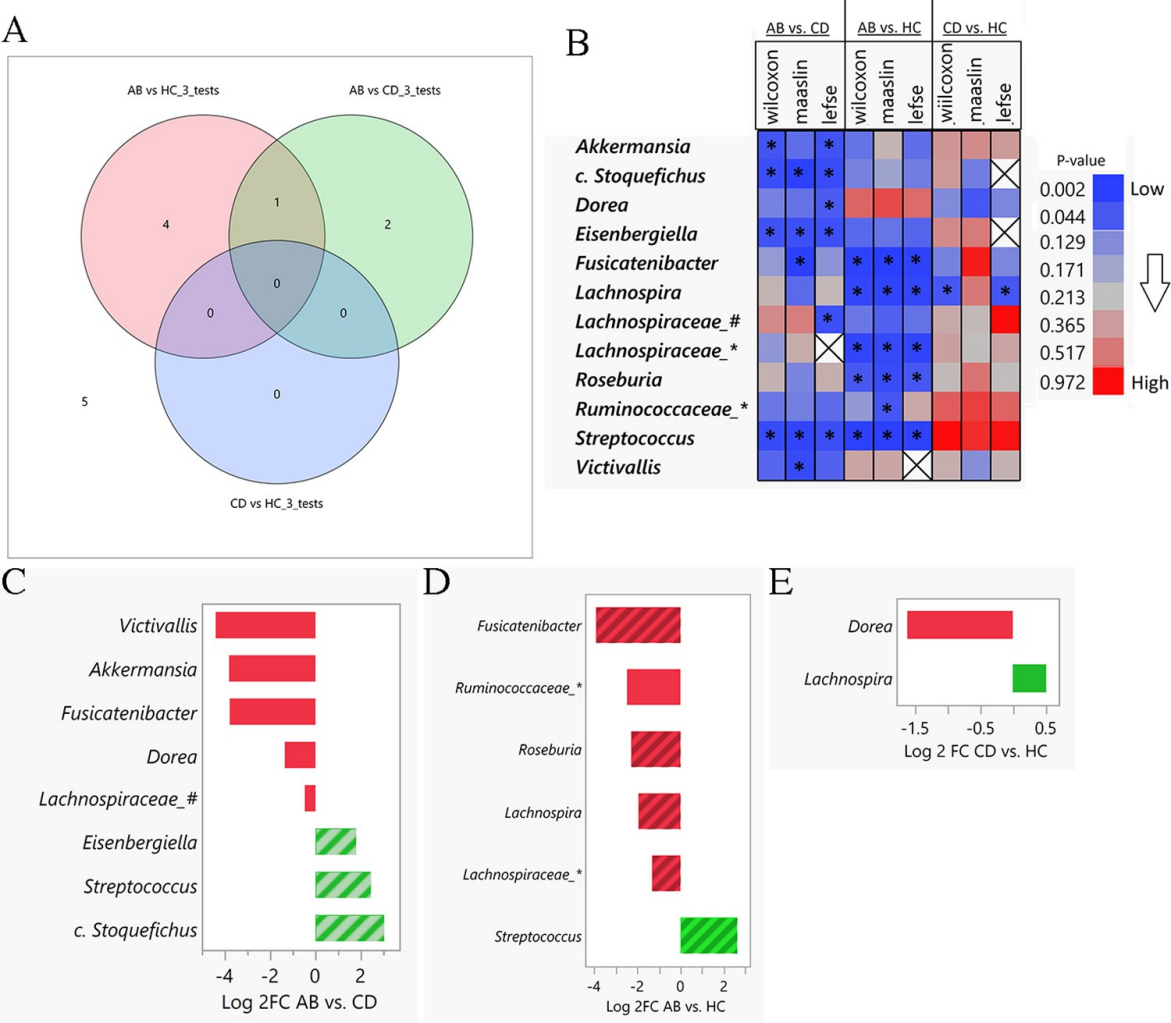
Differentially abundant taxa among groups. (**A**) Venn Diagram overlap comparing significant genera by all three tests (Wilcoxon, MaAsLin2, LEfSe) across each comparison. (**B**) Heat map of p-values for 12 significant genera (y-axis) in any of three testing procedures and group wise comparisons (x-axis). (* indicates *p* < .05 for comparison and test; X indicates that the genus was not assessed using LEfSe test procedure due to filtering). The color gradient shows the exact p-value to which the color corresponds to. Bar plot of **(C)** AB *vs*. CD, **(D)** AB *vs*. HC and **(E)** CD *vs*. HC Log2 fold change (x-axis) of genera (y-axis) found to be statistically different in comparison of interest. Shaded boxes indicate significant genus in all three test procedures. Neither taxon was significant by all three tests for CD *vs*. HC comparison. (Abbreviations: *c*. *Stoquefichus* = *Candidatus_Stoquefichus*; Ruminococcaceae_* = *Ruminococcaceae_UCG_014*; *Lachnospiraceae*_# = *Lachnospiraceae_FCS020_group*; *Lachnospiraceae*_* = *Lachnospiraceae_UCG_001*).

Seven taxa (in bold in **Table 3**) were found to be significantly different (*p* < .05) in at least one of the two group comparisons by all three tests. The overlap between the three pairwise comparisons of the seven taxa was assessed using a Venn Diagram analysis (**Fig 5A**). In addition, a color map showing *p*-values for all tests and group comparisons is shown in **Fig 5B**.

Eight genera were found to be different in AB *vs*. CD groups by any test. Three of those genera were significantly different and more abundant in AB in all tests: *Streptococcus*, *Eisenbergiella*, and *candidatus Stoquefichus* (genus candidate) (**Fig 5C**). Five genera were found to be different in ABs *vs*. HCs in all tests: *Streptococcus*, *Fusicatenibacter*, *Lachnospira*, *Lachnospiraceae_UCG_001*, and *Roseburia*. (**Fig 5D**). In CD *vs*. HC group comparison, two genera were found significant in at least one test (**Fig 5E**).

Results from each individual testing procedure are presented in **S1–S3 Figs**. Briefly, eight taxa were found to be significantly different using the Wilcoxon Sign Rank Sum test procedure (*p*<0.05) (**S1A Fig and S2 Table)** and the Venn Diagram overlap analysis is shown in **S1B Fig**. Heat trees are shown in **S1C–S1E Fig** (**S3C–S3E Table**). Results for LEfSe showed 10 genera to be statistically significantly different among any of the three comparisons *p* < .05. Each LDA effect score is plotted in **S2A–S2C Fig** for each group wise comparison. When considering group comparisons made using MaAsLin2, 10 significant taxa were found, and covariate plots were created to show the results from each test before and after covariate correction (**S3 Fig**).

### Gut untargeted metabolomics

**Fig 6:**
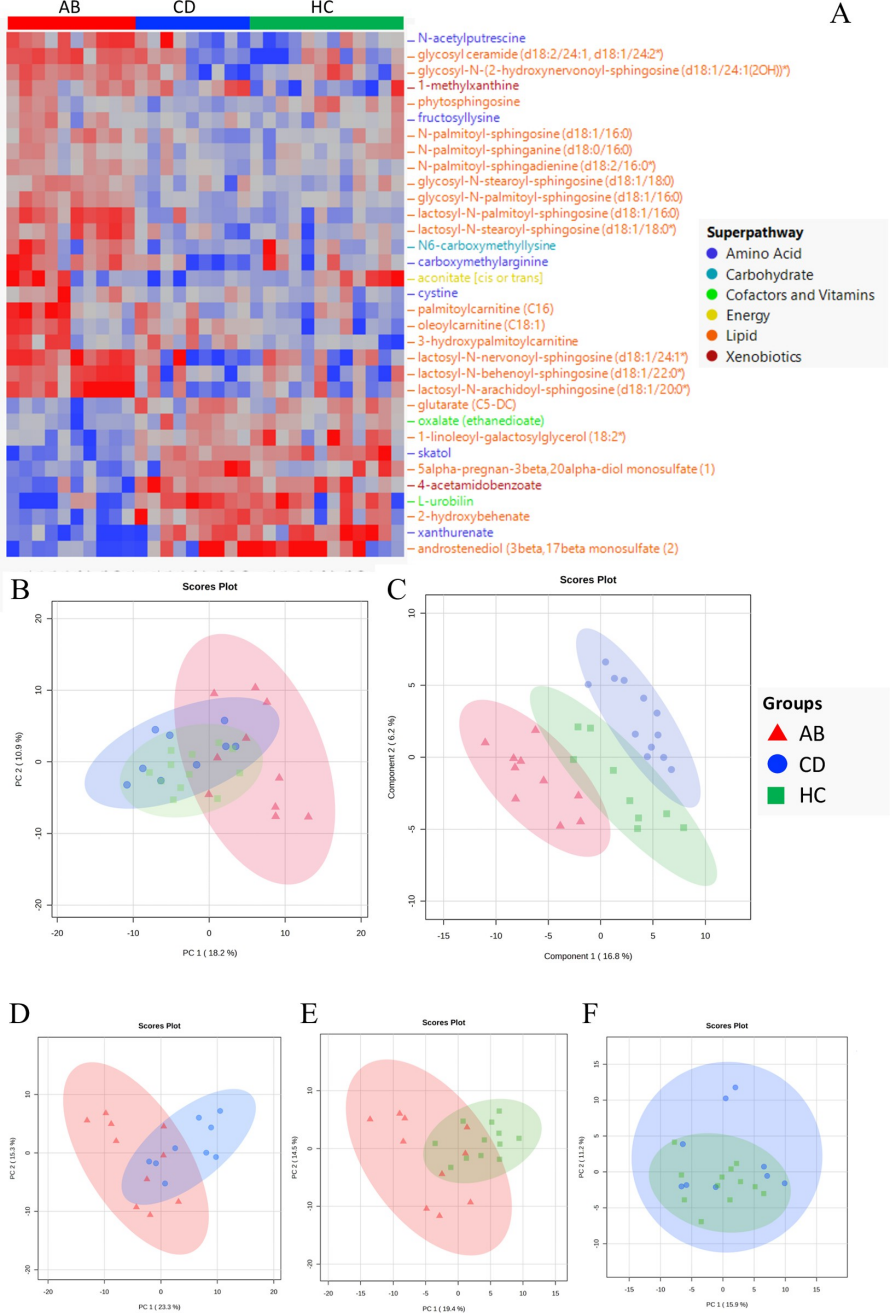
Metabolome differences among groups. (**A**) Hierarchical clustering of the 33 significantly different metabolites colored by super pathway among groups (*p* < .05, FDR corrected, Kruskal-Wallis test). Unsupervised (**B**) and supervised (**C**) ordination methods show separation between AB group *vs*. CD and HC groups (PCA), and AB, CD, and HC groups (PLSDA) respectively. The quality of the PLS-DA model was assessed by its ability to predict variance of the data with four components (Q_2_ = 0.575). Pairwise comparisons were observed by PCA in AB *vs*. CD (**D**), AB *vs*. HC (**E**), and CD *vs*. HC (**F**) comparisons. (AB: red, CD: blue, HC: green).

Untargeted metabolomic analysis identified 1032 metabolites. After excluding features with 50% or more missing values, a final set of 946 metabolites was analyzed and 33 differentially abundant metabolites were found among groups (**Fig 6A and S4 Table**). The top 30% differentially most abundant metabolites are shown in S4A Fig. Most of them (21/33) were lipids: Five fatty acid metabolites, five lactosylceramides, four hexosylceramides, and four ceramides, together with two steroids and one metabolite ascribed to sphingolipids synthesis. Different amino acids across groups include two biochemicals involved in tryptophan metabolism, one ascribed to lysine metabolism, one to cysteine metabolism, one to arginine metabolism, and one to polyamine metabolism. Most metabolites were more abundant in the AB individuals. Using PCA, AB group clustered separately from CD and HC groups (**Fig 6B**). With PLS-DA (**Fig 6C**), while observing a relative separation among groups, we found that the AB group showed the highest and lowest metabolite values (**S4B Fig**). Most metabolite differences were due to the AB group, with 28/33 (85%) metabolites significantly different in both the AB *vs*. CD and AB *vs*. HC comparisons. Pairwise comparisons (**Fig 6D–6F and S4 Table**) showed that amino acids belonging to tryptophan metabolism, including skatol and xanthurenate, were significantly less abundant in AB group compared to CD (*p* = .002 and *p* = 004, respectively) and HC (*p* < .001 and *p* < .001) groups. Biochemicals involved in lysine, cysteine, and arginine metabolism were more abundant in AB group than CD and HC groups, namely fructosyllysine (ABs *vs*. CDs, *p* < .001; ABs *vs*. HCs, *p* = .001), cystine (AB *vs*. CD, *p* = .002; AB *vs*. HC, *p <* .001), and carboxymethylarginine (ABs *vs*. CDs, *p* < .001; ABs *vs*. HCs *p* = .006). Differently, the two cofactors oxalate and l-urobilin, along with the xenobiotic 4-acetamidobenzoate, were less abundant among ABs than in CD and HC. Lastly, a xenobiotic belonging to xanthine metabolism (1-methylxanthine) and one carbohydrate (N6-carboxymethyllysine) were more abundant among AB individuals in both comparisons. Five metabolites exhibited significant differences in CDs *vs*. HCs comparison.

### Gut microbiome and metabolome associations

**Fig 7:**
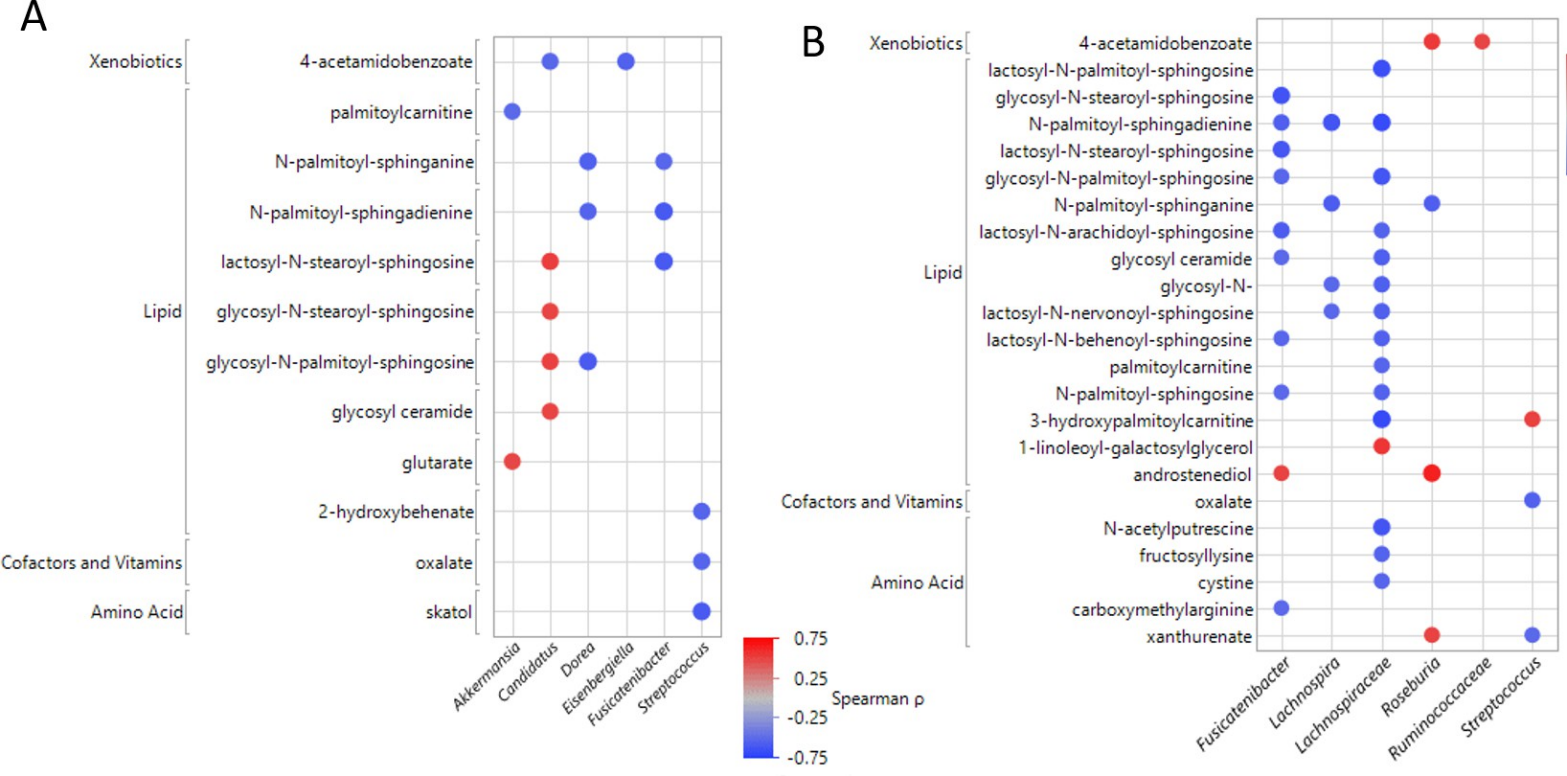
Associations between differential gut microbes and metabolites. **(A)** Differential AB *vs*. CD metabolites compared to microbes using Spearman correlation. (**B**) Differential AB *vs*. HC metabolites compared to microbes using Spearman correlation. Blue dot indicates negative correlation, red dot indicated positive correlation.

Differential features identified by fecal microbiome and metabolome analyses were correlated from pairwise comparisons (AB *vs*. CD, AB *vs*. HC, and CD *vs*. HC). When considering only those eight gut taxa found to be different between AB *vs*. CD groups from any test previously described and the 30 metabolites from this comparison, 17 significant correlations were identified. Six taxa were found to be significantly correlated with 12 metabolites (**Fig 7A**). *Streptococcus* negatively correlated with three metabolites from the vitamin/cofactors and amino acids while many associations were with metabolites derived from the lipid super pathway. There were two taxa showing positive associations with lipids: *Akkermansia* and *candidatus Stoquefichus*.

Likewise, in the AB *vs*. HC comparison, six taxa were correlated to 31 metabolites. This analysis revealed 37 significant associations (**Fig 7B**). The *Lachnospiraceae_UCG_001* genus showed associations with 15 metabolites and was negatively correlated with many lipid-related metabolites and some metabolites related to amino acid and vitamin/cofactor pathways. *Fusicatenibacter* genus was associated with ten metabolites from the lipid super pathway. There were seven microbiome/metabolite pairs that were positively associated and three of those were with the *Roseburia* genus. We didn’t any significant correlation when investigating CDs *vs*. HCs. In addition, we performed a multivariate analysis comparing *all* gut taxa to *all* gut metabolites in order to understand the relationships within each group (**S3 Table**). All by all Spearman correlations were computed within each group and a total of 149 significant correlation pairs were found within ABs (**S5A Fig**), 258 were found within the CD group (**S5B Fig**), and 33 within the HC group (**S5C Fig**).

### Other ancillary collected measures

No significant differences were observed in dietary intake across groups in total calorie intake (kcal) nor in percent calories from carbohydrate, protein, or fat (**S5 Table**). As anticipated, calories from alcohol were different among groups (*p*‘s < .001) with the CD individuals consuming the most.

The number of self-reported pre-existing medical conditions and concomitant medications was significantly different among the three groups (*p* = .004 and *p* = .028, respectively). Overall, ABs reported significantly more medical conditions and medications than CDs and HCs. AB individuals reported a total of 40 medical conditions, with a 4±1.8 per subject, while CD group reported 12 conditions (1.3±1.2 per subject) and HC group 19 conditions (1.6±1.3 per subject), adding up to 71 conditions reported (**Table 1 and S6A Fig**). These medical conditions were cross-checked with the list of diseases reported by Jackson *et al*., 37/71 (52.1%) conditions reported by our sample were found to be in common with the list; 21/40 (52.5%) conditions reported by ABs, 4/12 (33.3%) by CDs, and 12/19 (63.1%) by HCs overlapped with those listed by Jackson *et al*. (**S6B Fig**). ABs reported taking 22 medications (2.2±1.6 per participant), while CDs reported 6 (0.7±1.4 per participant) and HCs 8 medications (0.7±1.1 per participant), adding up to 36 medications taken by all (**Table 1 and S7A Fig**). When these medications were cross-checked with the list of medications by Jackson *et al*., 17/36 (47.2%) medications taken by our sample were found to be in common (11/22 (50%) by ABs, 3/6 (50%) by CDs, and 3/8 (37.5%) by HCs) overlapped (**S7B Fig**). A total of 37 diseases and 17 medications reported by the study participants across the AB, CD, and HC groups matched those found by Jackson *et al*. to have a significant association with a gut microbiome marker.

The gastrointestinal permeability assays showed inconsistencies in the participants’ fasting status of 16 participants; therefore, no conclusions were made using these data. However, sugar excretion curves over the sampling times (sucrose: **S8A Fig**, sucralose: **S8B Fig** and lactulose/mannitol ratio: **S8C Fig**) were plotted for the 15 participants who did not have sugar detected at the baseline samples.

## Discussion

This study reports the results of a case-control clinical investigation in an outpatient setting aimed at comparing the gut microbiome and metabolome among three groups of individuals: individuals with AUD who were treatment-seeking but newly abstinent (ABs), non-treatment-seeking individuals who were currently drinking (CDs), and healthy controls (HCs). All samples and measures were collected over approximately two weeks at the National Institutes of Health Clinical Center. In addition to fecal sample collection, other measures including addiction- and psychopathology-related phenotyping was carried out. The overall findings show that the healthy controls and the current drinkers did not differ much in the gut microbiome and metabolome features but that the abstinent individuals differed the most. Furthermore, the clinical phenotypic characterization showed that AB individuals had greater sleep disruption, poorer mental health status, and a higher number of medical comorbidities and concomitant medications. The greater differences observed in the AB group could be attributed to some biological processes going on in early abstinence after cessation of chronic alcohol use along with more severe AUD and comorbidities, a known characteristic of treatment-seeking (compared to nontreatment-seeking) individuals with AUD. Finally, while this experimental case-control study brings light to these facts, it is not possible to disentangle these two confounds, which could be contributing to the difference in AB individuals. Our findings are in contrast with other studies showing that individuals with AUD who abstain from alcohol may present with partial recovery of the gut microbiome [23, 32]. However, there are not enough studies nor enough studies with large sample sizes to conclude on the reversibility, or irreversibility, of alcohol-induced changes to the gut microbiota. Of note, other studies did not replicate these findings and found little or no changes in the gut microbiome after abstinence [7, 8, 10]. It bears mentioning that inpatient studies allow for a well-controlled, experimentally rigorous setting, but they are unable to control for other confounders such as changes in diet, general lifestyle habits and changes due to acute alcohol withdrawal. Additionally, inpatient studies allow for a well-controlled experimentally rigorous setting, but they are unable to control for other confounders such as changes in diet and general lifestyle habits and changes due to acute alcohol withdrawal. This current study provided an exemplary opportunity to capture gut microbiome samples in an outpatient setting allowing the participants to continue their normal daily lifestyle choices.

### Gut microbiome differences

The gut microbial diversity of observed ASVs and Chao1 indexes was significantly lower in the AB group compared to CD and HC groups; however, other diversity measures considering taxa relative abundance (*i*.*e*., Simpson and Shannon indexes) were not statistically different. In a study on the effects of moderate levels of alcohol consumption on the gut microbiome in both rats and humans, alcohol-treated rats had significantly lower species richness compared. Still, a human study looking at alcohol consumers did not display a similar alteration [62]. Firmicutes and Bacteroidetes phyla are dominant in human fecal samples, and previous animal and human studies point to increased Firmicutes following chronic heavy alcohol use [15–17, 27]. Taxonomic differences between groups were explored using a three-test procedure, finding seven taxa that passed all tests at a *p* < .05 for any comparison. Specifically, ABs presented a depletion of the beneficial genera *Akkermansia*, *Lachnospira*, *Roseburia*, *Fusicatenibacter*, and *Lachnospiraceae_UCG_001* and an increase in *Eisenbergiella*, *Streptococcus*, *and candidatus Stoquefichus*. These genera belong to the *Lachnospiraceae* family which have been implicated as part of the core of the gut microbiota, colonizing the gut lumen from birth. All members of this family are anaerobic, fermentative, and chemoorganotrophic, and some display strong hydrolyzing activities, for example through the activity of pectin methyl-esterase, α- and β-galactosidase, α- and β-glucosidase, and α-amylase [63]. *Lachnospiraceae* family plays a crucial role in the metabolism of carbohydrates, being able to utilize diet-derived polysaccharides, including starch, inulin, and arabinoxylan, to produce butyrate and other short-chain fatty acids (SCFAs). SCAFs have known, profound, beneficial effects on human health [64, 65]. In a previous study, *Eisenbergiella* has been shown to decrease after chronic alcohol drinking [66], which is discordant with what was found in this study, being more abundant in the AB group compared to CD and HC groups. Similarly, regarding the members of the *Lachnospiraceae* family, *Fusicatenibacter*, shown to be depleted in ABs compared to both CDs and HCs in this study, is an anaerobic, fermentative genus that supplies nutrients and energy to the host. Among the ABs, genera *Streptococcus* and *candidatus Stoquefichus* genera were more represented. Members of the *Streptococcus* genus are generally considered harmful and are common pathogens responsible for bacterial infections in individuals with AUD, especially if complicated by ALD [67]. *Candidatus Stoquefichus*, a-yet uncultured genus, has been found to be increased in the gut microbiota of a mouse model of obesity [68].

### Gut metabolomic differences

Fecal metabolic profiling of the study participants mirrored the results of the gut microbiome analysis. Both univariate and multivariate analyses showed the ABs’ fecal metabolome to be different from CDs and HCs. The 33 metabolites found to be significantly different among groups were primarily from the lipid and amino acid pathways. AB individuals had a lower abundance of tryptophan metabolites, such as skatol and xanthurenate. Tryptophan is an essential amino acid for protein synthesis that has emerged as a key player in the gut-brain axis. It is the only precursor for serotonin, which acts as a neurotransmitter in both the central and the enteric nervous systems [69, 70]. Additionally, tryptophan and its metabolites have a key role in maintaining gut-brain homeostasis; dysregulation of tryptophan metabolites plays a central role in the pathogenesis of many neuropsychiatric disorders, including AUD [71]. Gut bacteria influence tryptophan metabolism directly and indirectly, with corresponding changes in behavior and cognition. AB group, compared to CD and HC groups, had a higher abundance of metabolites of the lipid pathway, including lactosyl-N-palmitoyl-sphingosine, glycosyl-N-palmitoyl-sphingosine, and lactosyl-N-arachidoyl-sphingosine. Ingested triacylglycerides can be sequentially broken down to diacylglycerols (DAG), and then monoacylglycerols (MAG) [9] by the activity of secreted pancreatic lipases. The free fatty acids (FFAs) generated from each step, as well as the MAGs, can then be taken up by the epithelial cells of the intestinal lumen and repackaged into chylomicrons for distribution throughout the body (where at the tissue level they are broken down again to free fatty acids) [72]. As such, differences in DAGs, MAGs, and FFAs may indicate a difference in the action of lipases (bile secretion) and/or uptake of lipids. Lipids show an increase in various liver diseases, including alcohol-associated steatosis [73]. Given the known role of tryptophan and lipids in depression and other mental health disorders, we suggest that the differences in the gut metabolome observed in this study may be more reflective of the mental health comorbidities present among the AB group.

### Associations of the gut microbiome and metabolome

A correlation analysis to investigate the functional associations between differential gut taxa and metabolites was conducted. In the pairwise comparisons we found that *Streptococcus* genus, differentially more abundant among the ABs and found in higher abundance in individuals with AUD in other studies [10, 74, 75], negatively correlated with two tryptophan metabolism biochemicals, skatol and xanthurenate (ABs *vs*. CDs and AB *vs*. HCs comparisons, respectively). Conversely, *Roseburia* genus (less abundant in ABs when compared to HCs) and known SCFAs producer [76], was positively associated with xanthurenate metabolite. It has been showed that tryptophan metabolism pathways in the gut are differentially affected in diseases but remains interconnected and that these pathways are controlled by the gut microbiota [77]. The role played by *Streptococcus* and *Roseburia* genera still needs to be elucidated. *Candidatus Stoquefichus* (found in higher abundance among the ABs) exhibit positive correlations with all the hexosylceramides (more abundant in AB group compared to CD and HC groups). These metabolites are intermediates in sphingolipid metabolism that are involved in cell proliferation and differentiation, cell senescence, and apoptosis. Alteration of sphingolipid pathways contributed to alcohol-associated liver diseases [78, 79]. The differentially abundant ceramides were negatively associated with known SCAFs producers [80] *Fusicatenibacter* genus (AB *vs*. HC and AB *vs*. CD comparisons), *Dorea* (AB *vs*. CD comparison), *Roseburia*, *Lachnospira* and *Lachnospiraceae UCG 001* genera (AB *vs*. HC comparison). While this study does not allow to infer potential causal roles of these functional associations, future studies should investigate whether these gut microbiome-metabolome relationships may play a role in alcohol drinking and AUD.

### Clinical characteristics and differences across groups

There were no differences observed in demographic characteristics, blood marker liver tests, or in the FibroScan® evaluation in this study. Noteworthy, the liver markers AST and GGT were higher in the AB group at screening but not during the study (after the inpatient treatment period), showing how even short periods of abstinence may improve liver tests in people with AUD. When other clinical data was investigated, the AB individuals showed more severe traits related to AUD and psychological and mental health comorbidities than CDs and HCs. In fact, AB individuals had greater severity of AUD and significantly higher alcohol intake (pre-abstinence). Additionally, the AB group presented higher levels of depression and anxiety and poorer quality of sleep. These findings may partly explain the greater disruption of the gut microbiome and metabolome in AB individuals compared to CDs and HCs. The fact that the AB group (which were treatment-seekers) had clinically more severe characteristics, conditions, and medical and mental health comorbidities than CDs (non-treatment seekers) is consistent with previous studies examining treatment seeking and non-treatment seeking individuals with AUD [81–84]. Other potential explanations for the different gut microbiome and metabolome found among AB individuals may be attributed to the mental health differences observed. Previous studies have shown perturbations of the gut microbiome in multiple psychiatric disorders [4, 85, 86] as well as sleep disturbances [87]. A recent review and meta-analysis of 34 studies on the gut microbiome of individuals affected by various psychiatric disorders revealed a significant decrease in microbial community richness compared with healthy controls. It also showed no significant differences in diversity Shannon and Simpson indexes, in line with our findings. A transdiagnostic pattern of gut microbiota signatures was found: Depleted levels of *Faecalibacterium* and *Coprococcus* genera and enriched levels of *Eggerthella* genus were shared between major depressive disorder and anxiety, suggesting that these disorders are characterized by a reduction of anti-inflammatory, SCFA-producing bacteria, while pro-inflammatory ones are enriched.

Consistent with the conclusions above, AB group reported being affected by significantly more medical conditions and therefore taking significantly more medications than the other groups. Many of these conditions and medications overlapped with those that were found in the Jackson *et al*.’s study [14] to be associated with dysbiosis. These findings imply that gut microbiome and metabolome differences observed in AB individuals compared to CDs and HCs may therefore be due to a higher prevalence of diseases and a higher intake of medications in the AB group. This limits the ability to translate gut microbiome and metabolome findings from animal models to individuals with AUD, unless these variables are controlled for which was difficult to implement in this study due to the small sample size of the groups included.

### Dietary intake patterns across groups

Dietary intake showed no differences among groups during the two weeks of dietary intake assessment, other than in total alcohol intake, as expected. Some large population-wide studies have demonstrated that non-alcohol energy intake increases or remains unchanged with light and moderate drinking but decreases with heavy alcohol consumption to compensate for calories from alcohol [88]. Additionally, previous research has linked higher alcohol consumption with lower carbohydrate intake [89]. However, in this study, CD individuals consumed 14.4% of their energy from alcohol, whereas prior literature reports that the upper third of drinking Americans have an average of 20% of energy intake from alcohol [90]; this discrepancy could be due to CD group not having severe AUD than other studies. In addition to this, CDs may have underreported alcohol intake and other dietary intake variables. Furthermore, few studies have explored diet quality in abstinent and currently drinking individuals with AUD and more research needs to be conducted in this area to be able to better understand these findings.

This study presents a comprehensive gut microbiome and metabolome analysis, associated with a deep phenotyping of clinical and experimental measures of the three cohorts. The statistical strength of the study was limited due to the small groups’ sample size, and the lack of a pre-abstinence sampling timepoint preventing any longitudinal analyses. Additionally, the HC group was slightly overweight and reported some medication and pre-existing conditions which could have further confounded the comparisons. Additional long-term longitudinal outpatient studies are needed for better understanding of how prolonged abstinence (and lack of) may affect the gut microbial community.

## Conclusions

In conclusion, the present case-control study provides novel data on the gut microbiome and metabolome, along with a deep phenotyping characterization, in people with AUD from both newly abstinent treatment-seeking, non-treatment-seeking current drinkers, and matched controls. These individuals were investigated in their “real world” life across multiple outpatient visits, an important strength that gives more translational value to the findings described. Of note, however, our hypothesis was not proven. That is, we hypothesized gut microbiome/metabolome similarities between healthy controls and newly abstinent individuals with AUD, while the CD group was going to be different than the other two groups. By contrast, it was the AB group to separate itself, from a gut microbiome/metabolome standpoint, from the other two groups. The deep clinical phenotyping we conducted in this study allowed us to describe how the AB group had a more severe AUD history and more medical and psychiatric comorbidities. Therefore, all results considered, we speculate that the microbiome-metabolome differences here observed reflect mental health-related transdiagnostic traits and their sequelae (including more comorbidities and related medications) rather than an AUD-specific phenotype.

## Supporting information

S1 File (XLSX)

S2 File (DOCX)

S3 File (DOCX)

S1 Fig Overlapping taxa pairwise comparison between groups and heat trees. Pairwise comparison at the genus level between each group using Wilcoxon Test. (A) Dot plot of 9 genera (y-axis) found to be significant *(p* < .05, unadjusted) in pairwise comparison (x-axis). (B) Venn Diagram showing genera found to be significant that overlap between 3 pairwise tests. (C-E) The heat tree analysis leverages the hierarchical structure of taxonomic classifications to quantitatively (using the mean abundance) and statistically (using the non-parametric Wilcoxon Rank Sum test with *p* < .05) depict taxonomic differences between microbial communities. Red boxes show the end of the leaf (genus) in the heat tree for each plot. Taxa colored in red are more abundant in the AB (C, D) and CD (E) groups, taxa colored in blue are more abundant in the HC (C, E) and CD (D) groups. AB: Abstinent, CD: current drinkers, HC: healthy controls.
(TIF)

S2 Fig Lefse pairwise analysis. LEfSe LDA bar plots at the genus level of fecal microbial samples displaying the pairwise LDA scores between the three groups; abstinent group (AB), continuous alcohol drinking group (CD), and the healthy control group (HC). The bars represent the effect size (LDA) for a particular genus in a certain group. The length of the bar represents a log10 transformed LDA score. The heat maps show the LDA effect size from blue (negative) to red (positive). Shaded bars with an ‘*’ are genera that were found to be significant in the pairwise test (*p* < .05). (A) represents the pairwise comparison of AB to CD; (B) represents the pairwise comparison of AB to HC; and (C) represents the pairwise comparison of CD to HC. AB: Abstinent, CD: current drinkers, HC: healthy controls.
(TIF)

S3 Fig Results of covariate adjustment for MaAsLin2. Results of covariates adjustment (y axis) and without adjustment (x axis). P values for the comparisons between (A) AB and CD, (B) AB and HC, and (C) CD and HC groups did not improve with covariate adjustment (BMI and age), resulting in no significantly different taxa. AB: Abstinent, CD: current drinkers, HC: healthy controls.
(TIF)

S4 Fig Multivariate analysis of stool metabolome. Variable importance in prediction (VIP) scores extracted by PLS-DA of metabolites and heatmap of the average scaled expression values of the indicated VIP metabolites in all samples. The top 30 metabolites driving differences between group are represented (lowest VIP score = 2.261). Metabolites in AB group samples show the lowest (in most cases) or the highest abundance compared to HC and CD groups. AB: Abstinent, CD: current drinkers, HC: healthy controls.
(TIF)

S5 Fig Microbiome/metabolites correlations within groups. The correlations were computed within each group and a total of 149 significant correlation pairs were found within the ABs (A), 258 were found within the CDs (B), and 33 within the HCs (C). Most of the taxa within the AB group found to significantly correlate with metabolites, were represented in the CDs as well. Specifically, across the three comparisons, a high number of correlations were found in members from Lachnospiraceae and Oscillospiraceae families (Bacillota phylum): 62% in the CD group, 47% in the AB group, and 66% in the HC group. Streptococcus genus was found to be significantly more abundant in the AB group when compared to CD and HC groups. In the CD group Streptococcus genus was positively correlated with glyerolphosphoethanolamine (r = 0.950, p < .001), glycerophosphoserine (r = 0.917, p < .001), mannose (r = 0.917, p < .001), deoxycarnitine (r = 0.867, p = .002), and N,1, acetylspermidine (r = 0.850, p = .004). Conversely, in the same group we found negative correlations of Streptococcus with heptenedioate (C7:1-DC) (r = -0.865, p = .003), 2’-O-methylcytidine (r = -0.865, p = .003), and hexadecanedioate, C16 (r = -0.850, p = .004). In the AB group, *Streptococcus* genus correlated negatively with 7-ketocholesterol (r = -0.952, p < .001). *Lachnospira* genus, significantly less abundant in the AB group, was positively correlated with 3-hydroxyoctanoate (r = 0.931, p < .001) and with 1-palmitoyl-2-oleoyl-GPE (16:0/18:1) (r = 0.855, p = .002) in the AB group, while in the CD group correlated negatively with N-formylanthranilic acid (r = -0.900, p < .001), 2,hydroxybehenate (r = -0.900, p < .001), N-acetylglycine (r = -0.883, p = .002), urate (r = -0.867, p = .002), and pseudouridine (r = -0.850, p = .004). *Lachnospira* genus was positively correlated with indolepropionate (r = 0.867, p = .002) among the CDs. *Roseburia* genus, significantly less abundant in the AB group, correlated positively in the AB group with N,N,dimethylalanine (r = 0.867, p = .001), 12-ketolithocholate (r = 0.903, p < .001), and 3-dehydrodeoxycholate (r = .939, p < .001). The same genus was negatively associated with 3-(3-hydroxyphenyl) propionate (r = -0.883, p = .002), p-Cresol sulfate (r = -0.867, p = .002), and succinate (r = -0.850, p = .004) in the CD group. *Fusicatenibacter* genus, significantly less abundant in the AB group, negatively correlated with dibutyl sulfosuccinate (r = -0.865, p = .001) in the AB group. In the CD group this genus correlated negatively with Deoxycytidine monophosphate (dCMP) (r = -0.865, p = .003) and positively with methionine sulfoxide (r = 0.883, p = .002). Of note, we didn’t find any taxon/metabolite correlation passing the r± 0.85 cutoff in any group of *candidatus Stoqueficus* and *Eisenbergiella* (more abundant taxa among the ABs), as well as Lachnospiraceae_UCG_001 family, taxon depleted among ABs (Table 3).
(TIF)

S6 Fig Reported pre-existing conditions per participant and overlapping diseases with Jackson *et al*. (2018). A) Reported participant preexisting conditions use over entire patient population. Blue dots indicate condition of AB participants, red dots indicate condition of CD participants and green dots indicate condition of HC participants. B) Preexisting conditions that overlap with Jackson et al. (2018) when selecting at a 20% FDR in S5 Table (Jackson et al., 2018). (AB: Abstinent, CD: current drinkers, HC: healthy controls).
(TIF)

S7 Fig Reported medication taken by participant and overlapping medications with Jackson et al. (2018). A) Reported participant medication use over entire patient population. Blue dots indicate medication taken by AB participants, red dots indicate medication taken by CD participants and green dot indicates medication taken by HC participants. B) Medications that overlap with Jackson et al. (2018) when selecting at a 20% FDR in S4 Table (AB: Abstinent, CD: current drinkers, HC: healthy controls).
(TIF)

S8 Fig Gastrointestinal permeability curves. Detection of sucrose (A), Sucralose (B) and Lactulose/Mannitol ratio (C) by ultra-performance liquid chromatography mass spectrometry (UPLC-MS) analysis in suspected non-fasters participants. L/M ratio corresponded to the fractional excretion (FE) of lactulose and mannitol = (urine concentration from MS x total urine volume excreted)/sugar input). L/M ratio was calculated as FE lactulose/FE mannitol. Data are presented as mean values +/- SD. AB: Abstinent, CD: current drinkers, HC: healthy controls. Baseline = 40 minutes before starting the experiment, sampling times during the experiment: 1^st^ ≈ 90 min, 2^nd^ ≈ 100 min, 3^rd^ ≈ 180 min, 4^th^ ≈ 240 min, 5^th^ ≈ 280 min AB: Abstinent, CD: current drinkers, HC: healthy controls.
(TIF)

S1 Table (XLSX)

S2 Table (XLSX)

S3 Table (XLSX)

S4 Table (XLSX)

S5 Table (XLSX)
