# Supplementary material for: Gut microbial diversity and functional characterization in people with alcohol use disorder: A case-control study
Source: PLoS One. 2024 Jun 12;19(6):e0302195. doi: 10.1371/journal.pone.0302195 (PMC11168635; doi:10.1371/journal.pone.0302195)
Supplement: S3 File — (DOCX) [file pone.0302195.s003.docx]

**SupplementarY Methods**

***Study Inclusion Criteria***

*All Participants*

- Male or female individuals 21-70 years old (inclusive)

*Specific for Abstinent Group: AB*

- Current Alcohol Use Disorder (AUD) by DSM-5 criteria
- Being alcohol abstinent for at least 4 weeks with minimum of 2 weeks in a non-protective environment at the time of study screening

*Specific for Current Drinking: CD*

- Current Alcohol Use Disorder (AUD) by DSM-5 criteria
- Non-treatment seeking for alcohol use

Satisfying heavy drinking criteria during 4-weeks prior to screening “for men, >14 standard drinks in any one week **and** ≥4 drinks per occasion at least once per month over the past 30 days; for women, >7 drinks per week **and** ≥3 drinks per occasion at least once per month over the past 30 days” **and** any drinking during the 2-day prior to signing the study-specific consent.

*Specific for Healthy Controls: HC*

- No current or past diagnostic of AUD by DSM-5 criteria
- Non-alcohol drinkers or moderate alcohol drinkers i.e., up to 1 drink per day on average and not meeting NIAAA criteria for:
- heavy (i.e., for men, >14 standard drinks in any one week or ≥4 drinks per occasion at least once per month over the past 30 days; for women, >7 drinks per week or ≥3 drinks per occasion at least at least once per month over the past 30 days)
- or binge drinking (i.e., drinking 5 or more standard drinks on the same occasion on at least 1 day in the past 30 days for both male and female)

***Study Exclusion Criteria***

- Current pregnancy or lactation.

- Positive urine drug test for illegal drugs.

- Presence of active implantable electronic devices (e.g., defibrillators, pumps, pacemakers).

- The following current medical conditions: diabetes; chronic gut inflammatory diseases; gastrointestinal or any other type of cancer; short bowel syndrome; conditions requiring parenteral nutrition.

- Self-reported diarrhea or other symptoms of possible enteritis (past 7 days).

- Recent history of sigmoidoscopy or colonoscopy (past 30 days).

- Current use (past 90 days) of the following medications: oral and/or intravenous (IV) antimicrobials (specifically: antiviral, antifungal, or antibiotics); prebiotics; probiotics; laxatives; antispasmodic drugs; oral, intramuscular (IM) or IV steroids

- Any other reason or clinical condition that the Principal Investigator (PI) or Medical Advisory Investigator (MAI) considers unsafe for the individual or not in the best interest of the study research integrity.

***Alcohol-related clinical measures***

The Structured Clinical Interview for DSM-5 (SCID-5)(1) is a semi-structured interview administered to diagnose AUD. The number of endorsed criteria provides a diagnosis of AUD as mild (2 or 3 symptoms), moderate (4 or 5 symptoms), or severe (6 or more symptoms).

The Lifetime Drinking History (LDH)(2) is a self-administered questionnaire that assesses lifetime alcohol consumption during different periods of life. It was developed on the basis of a retrospective interview-based procedure(3). It is used to identify patterns of alcohol use, abuse, and dependence beginning with the onset of regular drinking and ending with the individual’s current drinking pattern. For each period, the subject reports the mean number of days per year he or she drank any amount of alcohol, and the mean number of drinks per drinking day. In the same way, binge drinking, defined as drinking ≥5 or more drinks for men or ≥4 drinks for women per occasion, is reported.

The Alcohol Use Disorders Identification Test (AUDIT)(4) is a 10-item self-report questionnaire that covers the domains of alcohol consumption, drinking behavior, and alcohol-related problems. It is a screening instrument for hazardous and harmful alcohol consumption. Responses to each question are scored from 0 to 4, giving a maximum possible score of 40. AUDIT scores ≥ 8 are recommended as indicators of hazardous and harmful alcohol use, as well as possible alcohol dependence.

The Alcohol Dependence Scale (ADS)(5, 6) is a 25-item self-administered questionnaire that provides an overall score by evaluating past-year psychiatric and physical symptoms of alcohol dependence, i.e., impaired control over drinking, awareness of a compulsion to drink, increased tolerance to alcohol, alcohol withdrawal symptoms, and salience of drink-seeking behavior. The ADS total score ranges from 0 to 47, with higher scores indicating more severe alcohol dependence. Specifically, scores of 1-13 suggest a low level, 14-21 an intermediate level, 22-30 a substantial level, and 31-47 a severe level of alcohol dependence, respectively. A score of 9 or more is considered an indicator of a current diagnosis of alcohol dependence. The ADS has demonstrated excellent predictive value with respect to the diagnosis of alcohol dependence and may provide additional contextual data on individualized clinical course characteristics of alcohol drinking behavior.

The Timeline FollowBack (TLFB)(7) questionnaire retrospectively assesses an individual’s daily alcohol intake in the 90 days prior to the interview date and yields several measures that provide a relatively accurate portrayal of an individual’s drinking amount, frequency, and pattern. This questionnaire estimates the amount of alcohol consumption expressed as number of “standard” drinking units (approximately 14 g pure alcohol), regardless of the type of alcohol beverages consumed. Several measures can be estimated from the TLFB, including the number of heavy drinking days and the average number of drinks per day over the last 90 days, which were used in the present study.

The Penn Alcohol Craving Scale (PACS)(8) is a 5-item questionnaire that measures an individual’s craving to drink alcohol in the past week. It includes questions about the frequency, intensity, and duration of craving, the ability to resist drinking, and an overall rating of craving.

The Clinical Institute Withdrawal Assessment of Alcohol Scale, Revised (CIWA-Ar)(9) is a 10-item scale geared towards objectifying alcohol withdrawal severity and guiding management decisions, such as the administration of benzodiazepines. The 10 items of the scale correspond to 10 common symptoms and signs of alcohol withdrawal, i.e., nausea and vomiting, tremor, paroxysmal sweats, anxiety, agitation, tactile disturbances, auditory disturbances, visual disturbances, headache, orientation, and clouded sensorium. An overall score <10 indicates minimal withdrawal, of 10-15 mild withdrawal, of 16-20 modest withdrawal, of >20 severe withdrawal. The maximum score is 67.

***Psychological measures***

The Pittsburgh Sleep Quality Index (PSQI) is 19-item self-report questionnaire designed to evaluate sleep quality and disturbances over a 1-month time interval. The 19 items generate seven "component" scores: subjective sleep quality, sleep latency, sleep duration, habitual sleep efficiency, sleep disturbances, use of sleeping medication, and daytime dysfunction. The sum of scores for these seven components yields one global score.

The Montgomery-Åsberg Depression Rating Scale (MADRS)(10) is the depression subscale of the Comprehensive Psychopathological Rating Scale (CPRS)(11). It is a 10-item self-administered questionnaire evaluating the core symptoms of depression: apparent sadness, reported sadness, inner tension, reduced sleep, reduced appetite, concentration difficulties, lassitude, inability to feel, pessimistic thoughts, suicidal thoughts. Participants rate each symptom on a Likert scale ranging from 0 to 6, with 0 indicating no presence of a symptom and 6 indicating continuous presence of a symptom. The overall score ranges from 0 to 60. Higher MADRS score indicates more severe depression. Usual cutoff points are 0 to 6 normal/symptom absent, 7 to 19 mild depression, 20 to 34 moderate depression, and 35 to 60 severe depression.

The Brief Scale for Anxiety (BSA)(12) is the anxiety subscale of the Comprehensive Psychopathological Rating Scale (CPRS)(11). It is a 10-item scale well established as a diagnostic self-assessment for anxiety. The BSA, being designed as a state measure, assesses current symptoms of somatic and psychological anxiety, both of which are present together in typical anxiety states. Four items (inner tension, hostile feelings, worrying over trifles, and phobias) are psychological symptoms of anxiety and five (hypochondriasis, autonomic disturbances, aches and pains, and muscular tension) are clear-cut somatic anxious symptoms. Reduced sleep is considered independently. The 10 items are each rated on a seven-point Likert scale, with a scoring range of 0-70. If there is particular interest in differentiating between the effects of psychological and somatic symptoms of anxiety, the scores for the psychological and somatic components can be analyzed separately.

The Profile of Mood States (POMS)(13) is a 65-item self-report questionnaire that assesses short-term mood states over the course of brief assessment periods. These mood states are understood to be transient and frequently fluctuating (they may fluctuate within minutes to days). It is designed to evaluate six distinct mood dimensions: depression-dejection, tension-anxiety, fatigue-inertia, anger-hostility, vigor-activity, and confusion-bewilderment. In the present study, we focused on the domains of depression-dejection (‘POMS depression’) and tension-anxiety (‘POMS anxiety’).

***Study Population Screening***
Thirty-five individuals were initially enrolled and 31 individuals were included in the final analysis. The four individuals not included in the analyses were removed as follows: one healthy control (HC) was excluded due to having clinical depression, one currently drinking (CD) individual was removed due to gut microbiome sequencing problems and two abstinent (AB) individuals were not included because one voluntarily dropped out from the study (after the 1st study visit), and the other had a positive BrAc for alcohol followed by blood alcohol test after the third study visit.

***Study Design***

A diagnosis of current or past substance (cannabis, opioid, hallucinogen, inhalant, stimulant and/or sedative) use, bipolar, or psychotic disorders was exclusionary. Non-exclusionary diagnoses included current or past tobacco use, depressive, anxiety, obsessive-compulsive, or post-traumatic stress disorders. Candidates screened under the NIAAA screening and natural history protocol (14-AA-0181; NCT02231840) and if eligible, were consented for this study (17-AA-0093; NCT03152760). At each visit, physical examination, 12-lead ECG, vital signs, and laboratory testing were performed ahead of study assessments to confirm that participants continued to meet enrollment criteria; a breathalyzer was used to measure breath alcohol concentration and ensure that the AB group maintained abstinence during study participation. At first visit, participants underwent transient liver elastography and met with trained nutrition staff for instruction on how to collect dietary intake information. All participants were asked to collect fecal samples at home at each bowel movement and additionally, fecal samples (“in-clinic” samples) were collected at any study visit, if the participant had a bowel movement during that visit. Participants were scheduled for a gastrointestinal permeability test at one of their visits. At the six visits, participants completed alcohol-related, psychological, and other clinical assessments. The last study visit included any remaining assessments and a final evaluation of the participants (Figure 1). Race, ethnicity, age, and BMI were collected at screening and confirmed at first study visit. Hematology, blood chemistry, coagulation panel, liver function tests, lipid panel, thyroid panel, and urinalysis were collected at screening and during study visits.

***Transient Liver Elastography***

Transient liver elastography was performed using FibroScan® (Echosens, France). FibroScan® is a non-invasive diagnostic ultrasound-based device used to quantify liver stiffness (hardness), a measure of fibrosis, and liver fatty changes, a measure of steatosis, which are present in a variety of liver diseases(14). The equipment consists of a 5-MHz ultrasound transducer coupled to a base with a vibratory axis. FibroScan® systems are equipped with two types of probes for adults: a medium (M) probe for use in most individuals (with skin-liver capsule distance <25 mm) and an extra-large (XL) probe for obese individuals (with skin-liver capsule distance <25 mm). FibroScan® is carried out with the participant lying supine and with the right arm fully extended. It provides two parameters, liver stiffness measurement (LSM, in kilopascals, kPa) and controlled attenuation parameter (CAP, in decibels/meter, dB/m), which assess the degree of liver fibrosis and steatosis, respectively(15). CAP and LSM measurements are carried out simultaneously, and the median values of ten valid measurements are used to quantify liver fibrosis and steatosis. The LSM is a measure of the speed of the shear wave that is generated by a push pulse as it passes through the liver tissue. The shear wave propagates faster in hard liver tissue than in soft liver tissue. LSM scores range from 1.5 to 75.0 kPa based on this property. Normal scores are usually between 2.0 and 7.0 kPa. Result higher than the normal range indicate liver fibrosis. The CAP is a measure of the attenuation of the ultrasound beam. The stronger the liver steatosis is, the more the ultrasound beam passing through the liver tissue is attenuated. CAP scores range from 100 dB/m to 400 dB/m based on this property. A score <38 dB/m means that the amount of liver steatosis is not higher than normal. Normal livers can have up to 5% of fatty changes in them. CAP and LSM correspond to the median values of 10 valid measurements. LSM scores range from 1.5 to 75.0 kPa, with normal values between 2.0-7.0 kPa. CAP scores range from 100 dB/m to 400 dB/m, with normal values <38 dB/m. Participants with a LSM score ≥17.6 kPa, suggestive of severe fibrosis or cirrhosis (16), were not allowed to proceed further in the study and were referred to appropriate clinical care.

***Fecal Sample Collection and Processing***

In terms of fecal sample collection, in-clinic samples were collected by participants using a collection hat, allowing feces separation from toilet water and urine, and enables the feces to be leveled with a sterile wooden spatula and stored in a feces container with screw cap (Cat#80.734.001, Sarstedt, Newton, NC). At-home samples were collected by study participants into two sterile, screw-capped tubes – one with and one without RNAlater – and frozen within 24 hours of collection in a home freezer, before being brought for analysis to the next scheduled study visit. After the fecal samples were collected from participants at each visit, they were brought to a uniform consistency using a sterile spatula. They were then divided into aliquots; specifically, for microbiome analysis, 500 µL of feces were extracted and placed in each of two 2 mL Cryotube vials, where 1 mL of RNAlater was added to each Cryotube vial. Each tube vial was vortexed to homogenize the samples. For metabolomic analysis, 250 µL of feces were extracted and placed in each of two 2 mL Cryotube vials; no RNAlater was added. All samples were immediately transferred to -80°C freezer for storage. No samples were excluded from the analysis.

***Gut Microbiome Extraction and Sequencing***

Genomic DNA was extracted from fecal samples with the MagAttract Microbial DNA Kit (Qiagen) using a custom automated protocol on the Hamilton Microlab Star. Both positive and negative controls (Zymo, Irvine, CA) were included in the DNA extraction process and the 16S rRNA gene sequence amplification was processed as previously described(17). Samples were thawed on ice and a 200 μl aliquot from the fecal sample was used as input for the kit following the manufacturer protocol. Cells were lysed by bead beating on the TissueLyser (Qiagen) at 20 Hz for 20 minutes and the final elution volume was 110 μl. DNA concentrations in the samples were determined with the Bioanalyzer 2100 DNA 1000 chip (Agilent, Santa Clara, CA). The hypervariable V3-V4 regions of the bacterial 16S rRNA gene were amplified with primers 319F and 806R, as previously described (18, 19). High-quality amplicon sequences were obtained on a Illumina HiSeq 2500 instrument modified to generate 300 bp paired-end reads (19). A total of five million reads were retained following chimera removal. Amplicon sequence variants (ASVs) were generated by DADA2 and taxonomically classified using the RDP Naïve Bayesian Classifier (20) trained with the SILVA v128 16S rRNA gene database(21). Negative controls generated a negligible amount of sequencing reads, whereas positive controls generated the expected mock community (17). ASVs, compared to the traditional operational taxonomic units (OTUs), enable a more accurate detection of gut microbial diversity (22).

***Gut metabolomic profiling***

Gut metabolomic profiling was performed by Metabolon Inc., Morrisville, NC, USA using untargeted ultra-performance liquid chromatography-tandem mass spectrometry (UPLC-MS/MS, Waters ACQUITY, Milford, MA). Each sample was assigned a unique identifier that was associated with the original source identifier. This identifier was used to track sample handling, tasks, and results. All samples were maintained at -80°C until processed. Samples were prepared using the automated MicroLab STAR® system (Hamilton Company, Franklin, MA). Several recovery standards were added prior to the first step in the extraction process for QA/QC purposes, as described in the following section. To remove protein and dissociate small molecules bound to protein or trapped in the precipitated protein matrix, proteins were precipitated with methanol under vigorous shaking for 2 minutes (Glen Mills GenoGrinder 2000), followed by centrifugation. The resulting extract was divided into five fractions: two for analysis by separate reverse phase (RP)/UPLC-MS/MS methods in both positive and negative ion mode electrospray ionization (ESI), one for analysis by hydrophilic interaction chromatography (HILIC)/UPLC-MS/MS in negative ion mode ESI, and one sample was reserved for backup. Samples were placed briefly on a TurboVap® (Zymark) to remove the organic solvent. The sample extracts were stored overnight under nitrogen before preparation for analysis.

***QA/QC for metabolomic analyses***

Several types of controls were analyzed in concert with the experimental samples: a pooled matrix sample generated by taking a small volume of each experimental sample (or alternatively, use of a pool of well-characterized human plasma) served as a technical replicate throughout the data set; extracted water samples served as process blanks; and a cocktail of QC standards that were carefully chosen not to interfere with the measurement of endogenous compounds were spiked into every analyzed sample, allowed instrument performance monitoring and aided chromatographic alignment. Instrument variability was determined by calculating the median relative standard deviation (RSD) for the standards that were added to each sample prior to injection into the mass spectrometers. Overall process variability was determined by calculating the median RSD for all endogenous metabolites (i.e., non-instrument standards) present in 100% of the pooled matrix samples. Experimental samples were randomized across the platform run with QC samples spaced evenly among the injections.

***Dietary Intake Assessment***

Records were analyzed using the 2016-2019 Nutrition Data Systems for Research (NDSR) software (University of Minnesota, Minneapolis, MN). The NDSR allowed us to obtain data on energy intake (total, calories from macronutrients, *i.e.,* carbohydrate, protein, and fat, and calories from alcohol in kilocalories [kcal]), as well as intake of specific foods and group of foods of interest (alcohol, fiber, dairy products, yogurt, coffee, tea). Alcohol and dietary fiber were measured in grams, whereas dairy products, yogurt, coffee, and tea in servings per day (servings/day). Food records deemed unreliable due to the participant not providing details for one or more eating occasions or for any other reasons at the discretion by nutrition staff were excluded from the analysis.

***Gastrointestinal Permeability test***

Gastrointestinal permeability was assessed using a single four-probe solution (sucrose, sucralose, lactulose, mannitol), with the excreted urine sugar ratios providing a measure of permeability (23). After a baseline urine collection, fasting subjects ingested a permeability test solution (100 mL solution containing sucrose [10 g/dL], lactulose [5 g/dL], mannitol [1 g/dL], and sucralose [0.1 g/dL]) and urine was collected for 5 h. Gastric absorption of sucrose occurs between 0 and 3 h after solution ingestion. Lactulose is absorbed in the small intestine between 3 and 5 h and mannitol is used to standardize surface area. Colonic permeability is typically assessed by sucralose excretion of >5 h. Analysis of the four sugars in urine was performed in duplicate. Quantification was achieved by ultra-performance liquid chromatography mass spectrometry utilizing a Thermo Scientific Vanquish UPLC with a Thermo ID-X mass spectrometer HESI-II electrospray source at 2500V in negative ion mode at a mass resolution of 60,000. The internal standard solution contained D6-sucralose, D6-sucrose, U-13C6-mannitol, and U-13C12-lactulose. Calibration standard stocks contained a mixture of sucralose (0.025-10 ug/mL), sucrose (0.25-100 ug/mL), lactulose (1.0-250 ug/mL) and mannitol (10-800 ug/mL). Calibration standard stocks or urine (50 uL) was mixed with 450 uL internal standards in ACN, vortexed and then centrifuged at 4 ℃, 1400 rpm for 15 minutes. The supernatant was transferred to LC-MS vial and maintained at 4 ℃ for analysis. Injection volume was 5 uL. Separations were performed on a Waters Cortecs HILIC 2.7 μm, 2.1x100mm column (Waters Corp., Milford, MA) maintained at 35 ⁰C. The separation used solvent A (70% H20, 30% ACN, 0.1% NH3) and solvent B (20% H20, 80% ACN, 0.1% NH3) at a flow rate of 325 μL/min. The gradient started at 100% solvent B for 0.5min, decreased to 40% B at 3.25 min and maintained for 1 min, then returned to 100% B at 4.5 min until 6.5 min. Quantitation was based on the M-H m/z and retention time. Analyte and the isotopologue internal standard M-H m/z are sucralose (395.0079, 401.0449), sucrose (341.1099, 327.1466) lactulose (341.1099, 353.11492), and mannitol (181.0714, 187.0919, respectively. The calibration curve had a R2>0.999 with 1/x weighting, meets FDA LC-MS guidelines for linearity and quantitation. Sucrose and sucralose were used to determine gastric and colonic permeability, respectively, while the lactulose/mannitol (L/M) ratio was used to assess small intestinal permeability (with mannitol used to standardize small intestine surface area). Urine was stored at -80°C until analysis. Ultra-performance liquid chromatography-mass spectrometry (UPLC-MS) analysis was performed in duplicate on a Thermo Scientific Vanquish UPLC-Thermo ID-X mass spectrometer HESI-II electrospray source at 2500V in negative ion mode at a mass resolution of 60,000. Each analyte was paired with its corresponding stable isotope internal standard: D_6_-sucrose, D_6_-sucralose, U-^13^C_12_-lactulose, and U-^13^C_6_-mannitol (). Results were the mean of two injections. Raw MS data were converted to sucrose or sucralose output (%) = [(urine concentration from MS x total urine volume excreted)/sugar input] x 100. L/M ratio corresponded to the fractional excretion (FE) of lactulose and mannitol = (urine concentration from MS x total urine volume excreted)/sugar input). L/M ratio was calculated as FE lactulose/FE mannitol.

**Supplementary TABLES**

**Supplementary Table 1**: Supplementary Table 1_meds_condition_recoding.xls
**Supplementary Table 2**: Supplementary Table 2_Microbiome_Pairwise_Comparisons.xls
**Supplementary Table 3**: Supplementary Table 3_Microbiome-Metabolites_Correlations.xls
**Supplementary Table 4**: Differential fecal metabolites among AB, CD, and HC groups

| **Pathway** | **Biochemical** | **K-W (H)** | **p_adj_** | ***P*-value**  **AB *vs*. CD** | ***P*-value**  **AB *vs*. HC** | ***P*-value**  **CD *vs*. HC** |
| --- | --- | --- | --- | --- | --- | --- |
| Lysine | Fructosyllysine | 14.846 | 0.044 | **<0.001↑** | **0.001↑** | 0.644 |
| Cysteine | cystine | 15.365 | 0.044 | **0.002↑** | **<0.001↑** | 0.166 |
| Polyamine | N-acetylputrescine | 13.841 | 0.044 | **0.005↑** | **<0.001↑** | 0.749 |
| Tryptophan | skatol | 14.978 | 0.044 | **0.002↓** | **<0.001↓** | 0.546 |
|  | xanthurenate | 13.641 | 0.044 | **0.004↓** | **<0.001↓** | 0.696 |
| Urea cycle; Arginine and Proline | carboxymethylarginine | 13.823 | 0.044 | **<0.001↑** | **0.006↑** | 0.166 |
| Advanced Glycation End-product | N6-carboxymethyllysine | 13.368 | 0.044 | **0.002↑** | **0.008↓** | 0.095 |
| Ascorbate and Aldarate | oxalate (ethanedioate) | 14.329 | 0.044 | **0.002↓** | **<0.001↓** | 0.546 |
| Hemoglobin and Porphyrin | L-urobilin | 13.832 | 0.044 | **<0.001↓** | **0.008↓** | 0.127 |
| TCA Cycle | aconitate [cis or trans] | 13.922 | 0.044 | **0.004↑** | 0.41 | **<0.001↓** |
| Androgenic Steroids | androstenediol (3beta,17beta) disulfate (2) | 12.999 | 0.046 | **0.006↓** | **<0.001↓** | 0.804 |
| Ceramides | N-palmitoyl-sphingadienine | 13.23 | 0.045 | **0.003↑** | **0.001↑** | 0.859 |
|  | N-palmitoyl-sphinganine | 13.46 | 0.044 | **0.002↑** | **0.004↑** | 0.127 |
|  | N-palmitoyl-sphingosine | 12.715 | 0.05 | **0.002↑** | **0.003↑** | 0.749 |
| Fatty Acid Metabolism | 2-hydroxybehenate | 13.598 | 0.044 | **<0.001↓** | **0.009↓** | 0.127 |
|  | 3-hydroxypalmitoylcarnitine | 14.066 | 0.044 | 0.307 | **<0.001↑** | **0.014↑** |
|  | glutarate (C5-DC) | 12.862 | 0.048 | **0.001↓** | **0.005↓** | 0.456 |
|  | oleoylcarnitine (C18:1) | 13.78 | 0.044 | 0.206 | **0.002↑** | **0.004↑** |
|  | palmitoylcarnitine (C16) | 15.042 | 0.044 | **0.008↑** | **<0.001↑** | 0.214 |
| Galactosyl Glycerolipids | 1-linoleoyl-galactosylglycerol (18:2*) | 13.352 | 0.044 | **0.001↓** | **0.003↓** | 0.456 |
| Hexosylceramides (HCER) | glycosyl ceramide | 14.637 | 0.044 | **0.002↑** | **<0.001↑** | 0.546 |
|  | glycosyl-N-(2-hydroxynervonoyl-sphingosine *) | 15.926 | 0.044 | **<0.001↑** | **0.002↑** | 0.214 |
|  | glycosyl-N-palmitoyl-sphingosine | 17.637 | 0.044 | **<0.001↑** | **<0.001↑** | 0.644 |
|  | glycosyl-N-stearoyl-sphingosine | 15.453 | 0.044 | **<0.001↑** | **<0.001↑** | 0.859 |
| Lactosylceramides (LCER) | lactosyl-N-arachidoyl-sphingosine | 16.969 | 0.044 | **<0.001↑** | **<0.001↑** | 0.594 |
|  | lactosyl-N-behenoyl-sphingosine | 15.249 | 0.044 | **0.001↑** | **<0.001↑** | 0.644 |
|  | lactosyl-N-nervonoyl-sphingosine | 16.496 | 0.044 | **0.002↑** | **<0.001↑** | 0.127 |
|  | lactosyl-N-palmitoyl-sphingosine | 17.548 | 0.044 | **<0.001↑** | **<0.001↑** | 0.859 |
|  | lactosyl-N-stearoyl-sphingosine | 13.523 | 0.044 | **0.004↑** | **<0.001↑** | 0.859 |
| Progestin Steroids | 5alpha-pregnan-3beta,20alpha-diol monosulfate | 12.996 | 0.046 | **0.002↓** | **0.011↓** | 0.06 |
| Sphingolipid Synthesis | phytosphingosine | 14.44 | 0.044 | **<0.001↑** | 0.156 | **0.006↓** |
| Chemical | 4-acetamidobenzoate | 13.101 | 0.046 | **0.001↓** | **0.005↓** | 0.337 |
| Xanthine Metabolism | 1-methylxanthine | 13.695 | 0.044 | 0.348**↑** | **<0.001↑** | **0.008↑** |

Bold shows metabolite passing multiple comparisons correction ***p*<.05**. Arrows labeled as ↑ and ↓ represents minuend direction of change. K-W: Kruskal-Wallis

**Supplementary Table 5:** Dietary Intake Averages Across Groups

**Supplementary Figures**

**Supplementary Figure 1:** Overlapping Taxa Pairwise Comparison between groups and Heat Trees


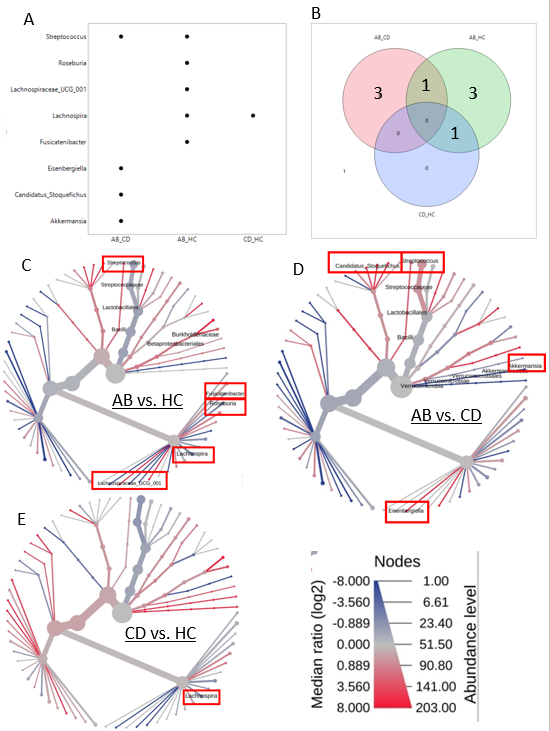


**Legend:** Pairwise comparison at the genus level between each group using Wilcoxon Test. (A) Dot plot of 9 genera (y-axis) found to be significant *(p*<.05, unadjusted) in pairwise comparison (x-axis). (B) Venn Diagram showing genera found to be significant that overlap between 3 pairwise tests. (C-E) The heat tree analysis leverages the hierarchical structure of taxonomic classifications to quantitatively (using the mean abundance) and statistically (using the non-parametric Wilcoxon Rank Sum test with *p*<.05) depict taxonomic differences between microbial communities. Red boxes show the end of the leaf (genus) in the heat tree for each plot. Taxa colored in red are more abundant in the AB (C, D) and CD (E) groups, taxa colored in blue are more abundant in the HC (C, E) and CD (D) groups. AB: Abstinent, CD: current drinkers, HC: healthy controls.

**Supplementary Figure 2:** Lefse pairwise analysis

**
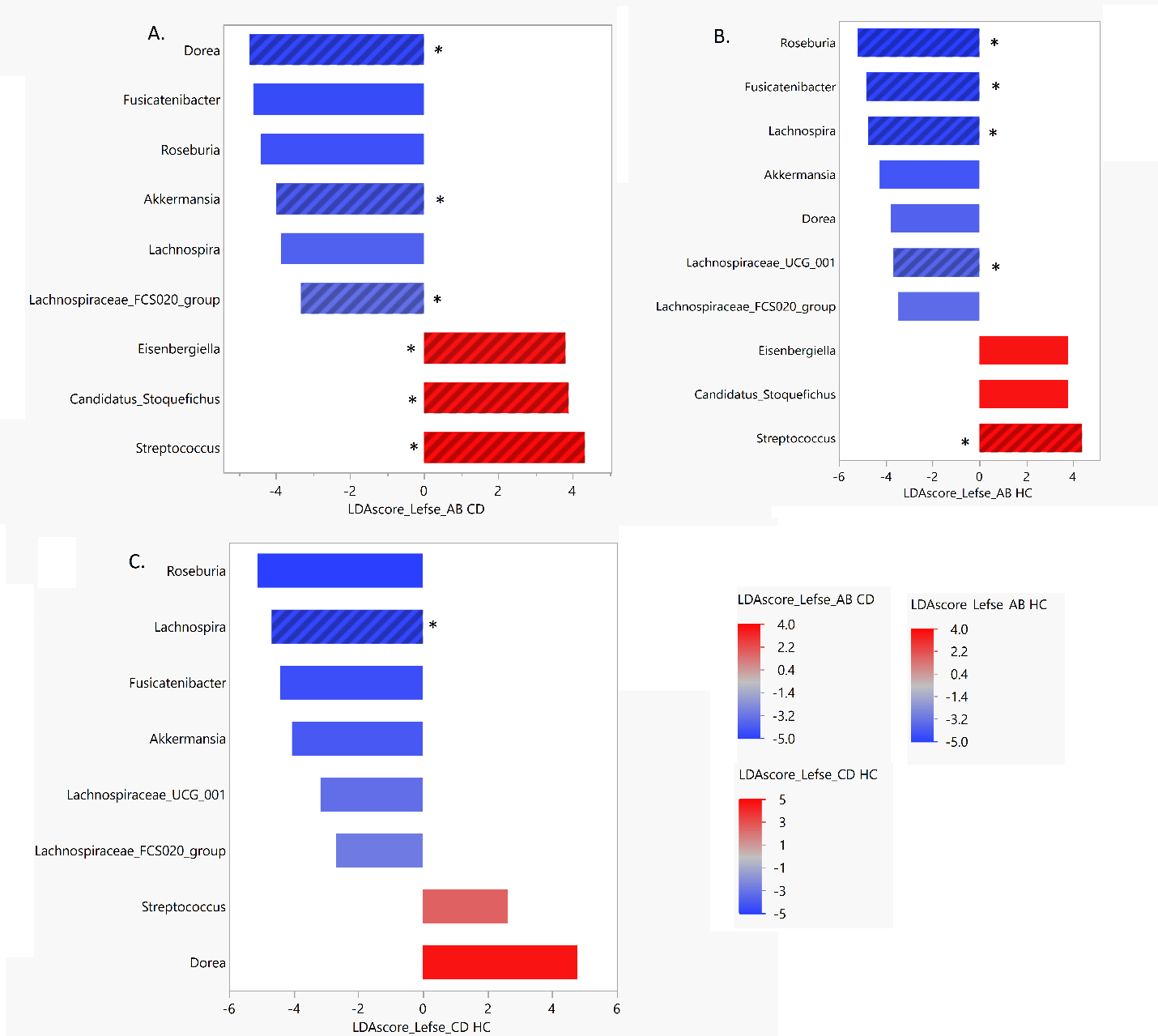
**

**Legend:** LEfSe LDA bar plots at the genus level of fecal microbial samples displaying the pairwise LDA scores between the three groups; abstinent group (AB), continuous alcohol drinking group (CD), and the healthy control group (HC). The bars represent the effect size (LDA) for a particular genus in a certain group. The length of the bar represents a log10 transformed LDA score. The heat maps show the LDA effect size from blue (negative) to red (positive). Shaded bars with an ‘*’ are genera that were found to be significant in the pairwise test (*p*<.05). (A) represents the pairwise comparison of AB to CD; (B) represents the pairwise comparison of AB to HC; and (C) represents the pairwise comparison of CD to HC. AB: Abstinent, CD: current drinkers, HC: healthy controls.

**Supplementary Figure 3**: Results of Covariate adjustment for MaAsLin2

**Legend:** Results of covariates adjustment (y axis) and without adjustment (x axis). *P* values for the comparisons between (A) AB and CD, (B) AB and HC, and (C) CD and HC groups did not improve with covariate adjustment (BMI and age), resulting in no significantly different taxa. AB: Abstinent, CD: current drinkers, HC: healthy controls.

**Supplementary Figure 4:** Top 30% Differential Metabolites and Multivariate Analysis of stool metabolome

A.

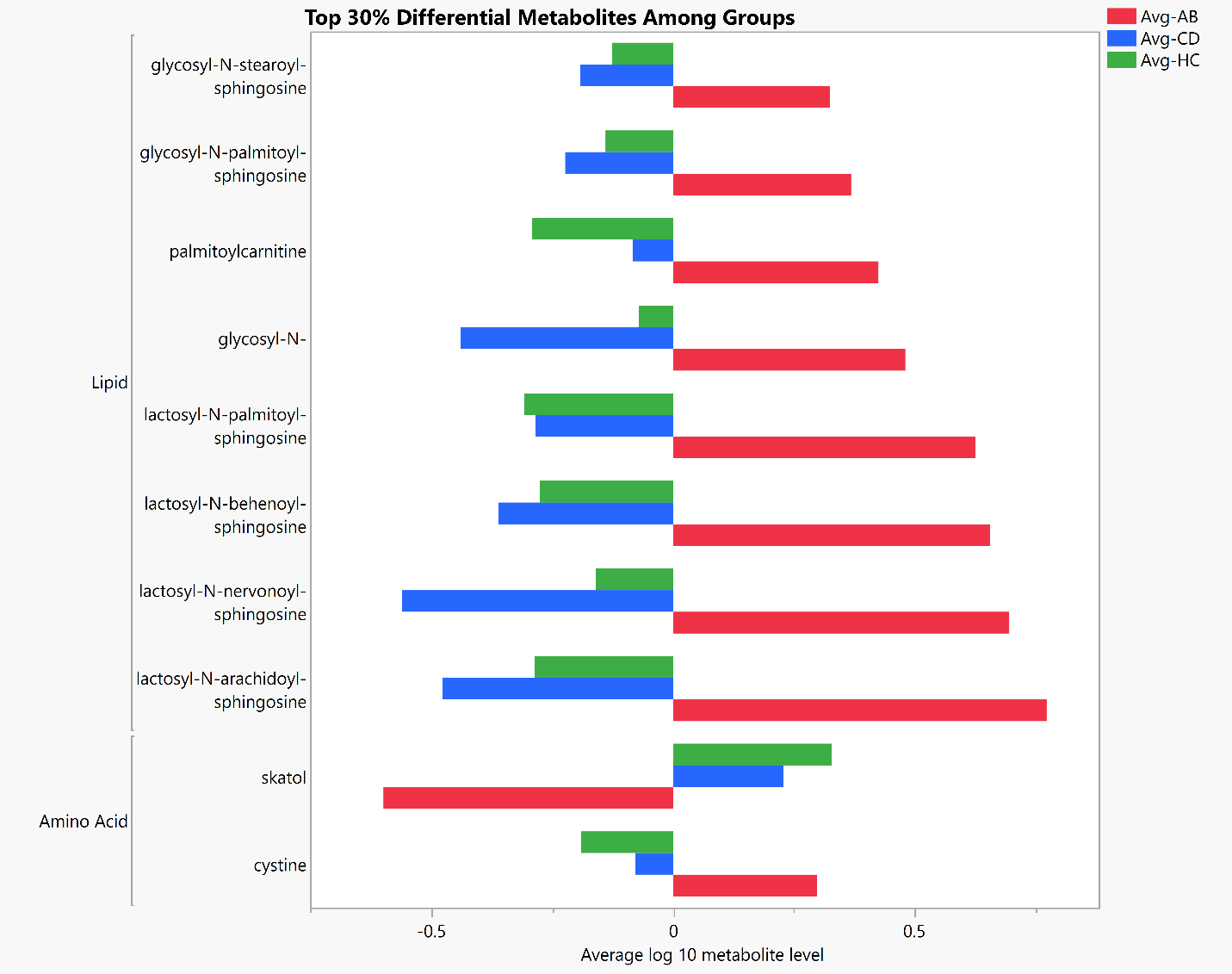


B.


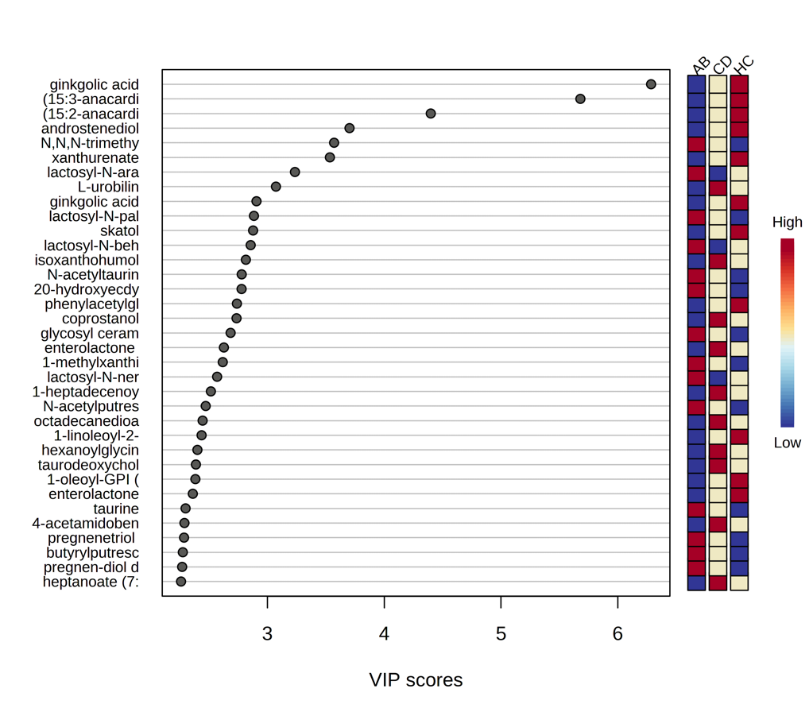


**Legend**: A) Top 30% differential metabolites among groups. B) Variable importance in prediction (VIP) scores extracted by PLS-DA of metabolites and heatmap of the average scaled expression values of the indicated VIP metabolites in all samples. The top 30 metabolites driving differences between group are represented (lowest VIP score =2.261). Metabolites in AB group samples show the lowest (in most cases) or the highest abundance compared to HC and CD groups. AB: Abstinent, CD: current drinkers, HC: healthy controls.

**Supplementary Figure 5**. Microbiome/metabolites correlations within groups


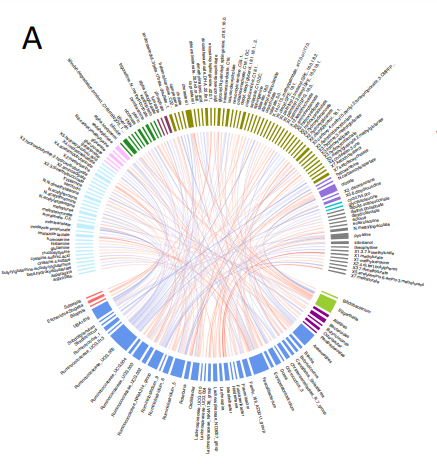


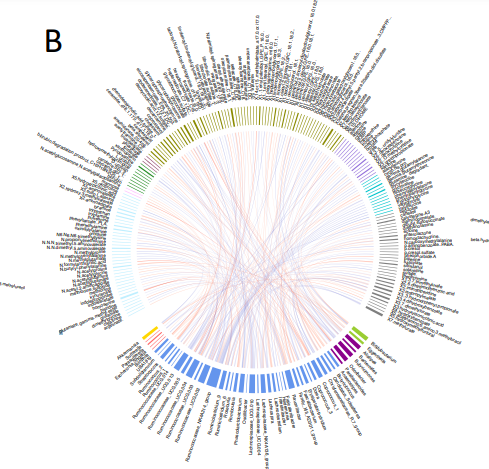


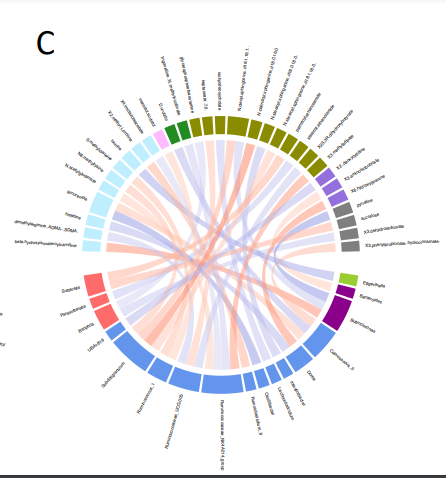

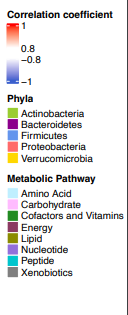


**Legend:** The correlations were computed within each group and a total of 149 significant correlation pairs were found within the ABs (**A**), 258 were found within the CDs (**B**), and 33 within the HCs (**C**). Most of the taxa within the AB group found to significantly correlate with metabolites, were represented in the CDs as well. Specifically, across the three comparisons, a high number of correlations were found in members from *Lachnospiraceae* and *Oscillospiraceae* families (Bacillota phylum): 62% in the CD group, 47% in the AB group, and 66% in the HC group. *Streptococcus* genus was found to be significantly more abundant in the AB group when compared to CD and HC groups. In the CD group *Streptococcus* genus was positively correlated with glyerolphosphoethanolamine (r=0.950, *p*<.001), glycerophosphoserine (r=0.917, *p*<.001), mannose (r=0.917, *p*<.001), deoxycarnitine (r=0.867, *p*=.002), and N,1,acetylspermidine (r=0.850, *p*=.004). Conversely, in the same group we found negative correlations of *Streptococcus* with heptenedioate (C7:1-DC) (r=-0.865, *p*=.003), 2'-O-methylcytidine (r=-0.865, *p*=.003), and hexadecanedioate,C16 (r=-0.850, *p*=.004). In the AB group, *Streptococcus* correlated negatively with 7-ketocholesterol (r=-0.952, *p*<.001). *Lachnospira*, significantly less abundant in the AB group, was positively correlated with 3-hydroxyoctanoate (r=0.931, *p*<.001) and with 1-palmitoyl-2-oleoyl-GPE (16:0/18:1) (r=0.855, *p*=.002) in the AB group, while in the CD group correlated negatively with N-formylanthranilic acid (r=-0.900, *p*<.001), 2,hydroxybehenate (r=-0.900, *p*<.001), N-acetylglycine (r=-0.883, *p*=.002), urate (r=-0.867, *p*=.002), and pseudouridine (r=-0.850, *p*=.004). *Lachnospira* was positively correlated with indolepropionate (r=0.867, *p*=.002) among the CDs. *Roseburia* genus, significantly less abundant in the AB group, correlated positively in the AB group with N,N,dimethylalanine (r=0.867, *p*=.001), 12-ketolithocholate (r=0.903, *p*<.001), and 3-dehydrodeoxycholate (r=.939, *p*<.001). The same genus was negatively associated with 3-(3-hydroxyphenyl)propionate (r=-0.883, *p*=.002), p-Cresol sulfate (r=-0.867, *p*=.002), and succinate (r=-0.850, *p*=.004) in the CD group. *Fusicatenibacter*, significantly less abundant in the AB group, negatively correlated with dibutyl sulfosuccinate (r=-0.865, *p*=.001) in the AB group. In the CD group this genus correlated negatively with Deoxycytidine monophosphate (dCMP) (r=-0.865, *p*=.003) and positively with methionine sulfoxide (r=0.883, *p*=.002). Of note, we didn’t find any taxon/metabolite correlation passing the r± 0.85 cutoff in any group of *candidatus Stoqueficus and* *Eisenbergiella* (more abundant taxa among the ABs), as well as *Lachnospiraceae*_UCG_001 family, taxon depleted among ABs (**Table 3**).

**Supplementary Figure 6:** Reported pre-existing conditions per participant and overlapping diseases with Jackson *et al.* (2018).


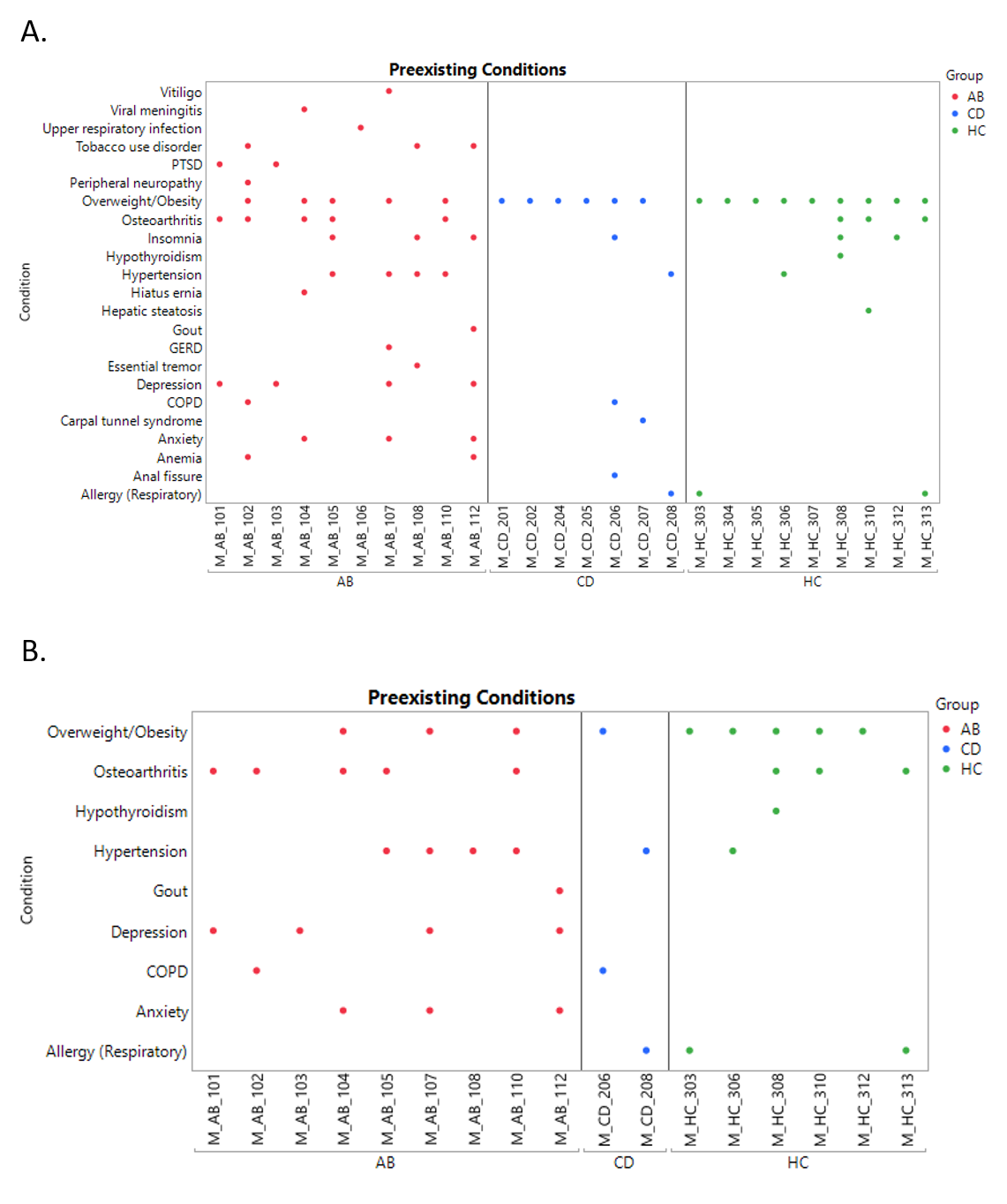


**Legend**: A) Reported participant preexisting conditions use over entire patient population. Blue dots indicate condition of AB participants, red dots indicate condition of CD participants and green dots indicate condition of HC participants. B) Preexisting conditions that overlap with Jackson et al. (2018) when selecting at a 20% FDR in Supplementary Table 5 (Jackson *et al.,* 2018). (AB: Abstinent, CD: current drinkers, HC: healthy controls).

**Supplementary Figure 7:** Reported medication taken by participant and overlapping medications with Jackson *et al.* (2018).

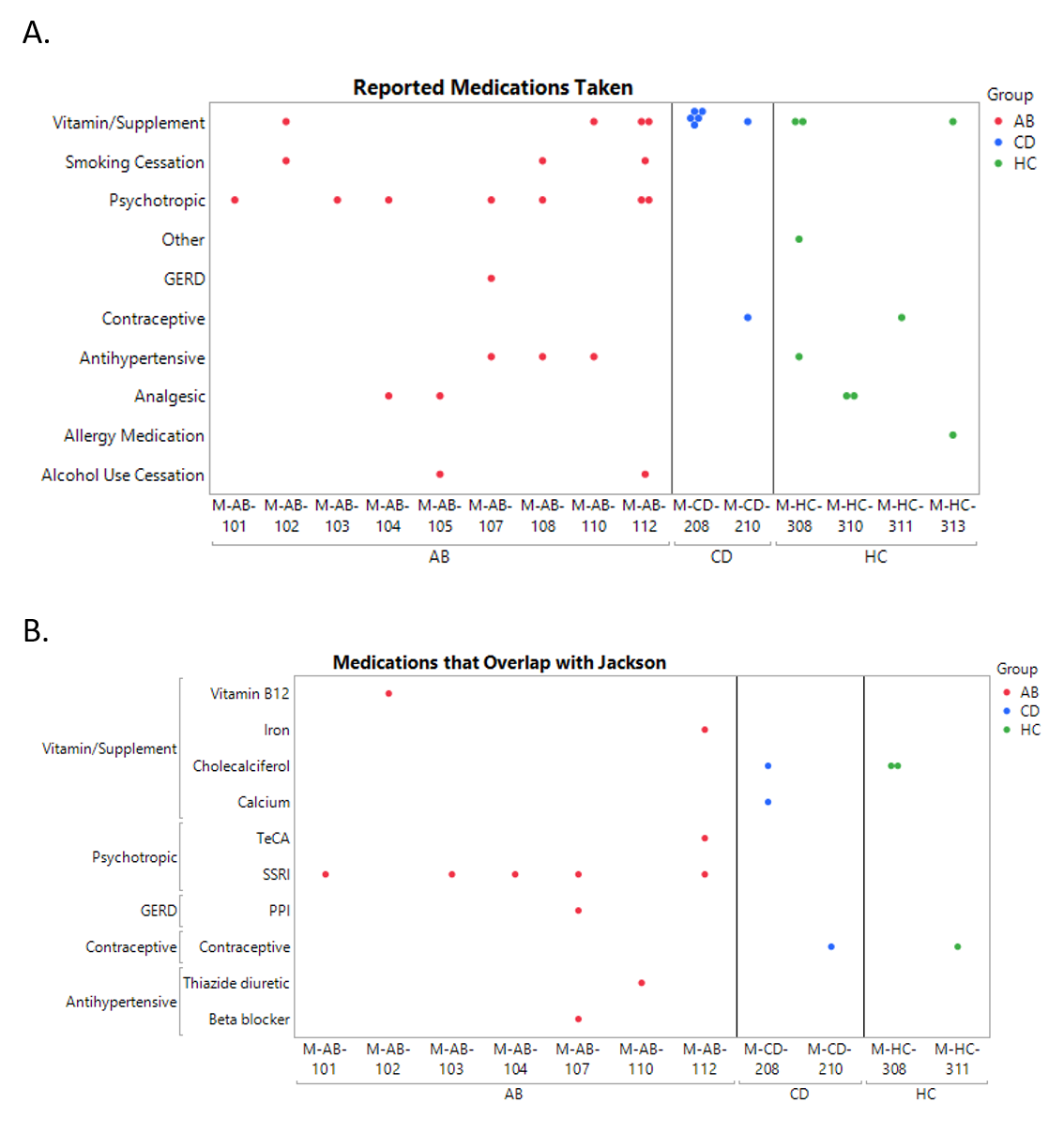


**Legend**: A) Reported participant medication use over entire patient population. Blue dots indicate medication taken by AB participants, red dots indicate medication taken by CD participants and green dot indicates medication taken by HC participants. B) Medications that overlap with Jackson et al. (2018) when selecting at a 20% FDR in Supplementary Table 4 (AB: Abstinent, CD: current drinkers, HC: healthy controls).

**Supplementary Figure 8**: Gastrointestinal permeability curves.

**Legend**: Detection of sucrose (A), Sucralose (B) and Lactulose/Mannitol ratio (C) by ultra-performance liquid chromatography mass spectrometry (UPLC-MS) analysis in suspected non-fasters participants. L/M ratio corresponded to the fractional excretion (FE) of lactulose and mannitol = (urine concentration from MS x total urine volume excreted)/sugar input). L/M ratio was calculated as FE lactulose/FE mannitol. Data are presented as mean values +/- SD. AB: Abstinent, CD: current drinkers, HC: healthy controls. Baseline = 40 minutes before starting the experiment, sampling times during the experiment: 1^st^ ≈ 90 min, 2^nd^ ≈ 100 min, 3^rd^ ≈ 180 min, 4^th^ ≈ 240 min, 5^th^ ≈ 280 min. AB: Abstinent, CD: current drinkers, HC: healthy controls.

**References**:

1. Michael First JW, Rhonda Karg, Robert Spitzer, American Psychiatric Association. *Structured Clinical Interview for DSM-5—Research Version (SCID-5 for DSM-5, Research Version; SCID-5-RV).* Arlington, VA; 2015.

2. Lemmens PH, Volovics L, and Haan YD. Measurement of lifetime exposure to alcohol: data quality of a self-administered questionnaire and impact on risk assessment. *Contemporary Drug Problems.* 1997;24(3):581-600.

3. Skinner HA, and Sheu WJ. Reliability of alcohol use indices. The Lifetime Drinking History and the MAST. *J Stud Alcohol.* 1982;43(11):1157-70.

4. Babor TF, Higgins-Biddle JC, Saunders JB, Monteiro MG, and Organization WH. World Health Organization; 1989.

5. Skinner HA, and Allen BA. Alcohol dependence syndrome: Measurement and validation. *Journal of Abnormal Psychology.* 1982;91(3):199-209.

6. Skinner HA, and Horn JL. *Alcohol dependence scale (ADS): User's guide.* Addiction Research Foundation; 1984.

7. Sobell LC, and Sobell MB. *Measuring alcohol consumption: Psychosocial and biochemical methods*. Totowa, NJ, US: Humana Press/Springer Nature; 1992:41-72.

8. Flannery B, Volpicelli J, and Pettinati H. Psychometric properties of the Penn alcohol craving scale. *Alcoholism: Clinical and Experimental Research.* 1999;23(8):1289-95.

9. Sullivan JT, Sykora K, Schneiderman J, Naranjo CA, and Sellers EM. Assessment of alcohol withdrawal: the revised clinical institute withdrawal assessment for alcohol scale (CIWA-Ar). *Br J Addict.* 1989;84(11):1353-7.

10. Montgomery SA, and Asberg M. A new depression scale designed to be sensitive to change. *Br J Psychiatry.* 1979;134:382-9.

11. Asberg M, Montgomery SA, Perris C, Schalling D, and Sedvall G. A comprehensive psychopathological rating scale. *Acta Psychiatr Scand Suppl.* 1978(271):5-27.

12. Tyrer P, Owen RT, and Cicchetti DV. The brief scale for anxiety: a subdivision of the comprehensive psychopathological rating scale. *J Neurol Neurosurg Psychiatry.* 1984;47(9):970-5.

13. Lorr M, McNair D, and Droppleman L. Manual: profile of mood states. *San Diego, CA: Educational and Industrial Testing Service.* 1971.

14. Afdhal NH. Fibroscan (transient elastography) for the measurement of liver fibrosis. *Gastroenterol Hepatol (N Y).* 2012;8(9):605-7.

15. Oeda S, Tanaka K, Oshima A, Matsumoto Y, Sueoka E, and Takahashi H. Diagnostic Accuracy of FibroScan and Factors Affecting Measurements. *Diagnostics (Basel).* 2020;10(11).

16. Foucher J, Chanteloup E, Vergniol J, Castéra L, Le Bail B, Adhoute X, et al. Diagnosis of cirrhosis by transient elastography (FibroScan): a prospective study. *Gut.* 2006;55(3):403-8.

17. Prochazkova P, Roubalova R, Dvorak J, Kreisinger J, Hill M, Tlaskalova-Hogenova H, et al. The intestinal microbiota and metabolites in patients with anorexia nervosa. *Gut Microbes.* 2021;13(1):1-25.

18. Fadrosh DW, Ma B, Gajer P, Sengamalay N, Ott S, Brotman RM, et al. An improved dual-indexing approach for multiplexed 16S rRNA gene sequencing on the Illumina MiSeq platform. *Microbiome.* 2014;2(1):6.

19. Holm JB, Humphrys MS, Robinson CK, Settles ML, Ott S, Fu L, et al. Ultrahigh-Throughput Multiplexing and Sequencing of >500-Base-Pair Amplicon Regions on the Illumina HiSeq 2500 Platform. *mSystems.* 2019;4(1).

20. Wang Q, Garrity GM, Tiedje JM, and Cole JR. Naive Bayesian classifier for rapid assignment of rRNA sequences into the new bacterial taxonomy. *Appl Environ Microbiol.* 2007;73(16):5261-7.

21. Quast C, Pruesse E, Yilmaz P, Gerken J, Schweer T, Yarza P, et al. The SILVA ribosomal RNA gene database project: improved data processing and web-based tools. *Nucleic Acids Res.* 2013;41(Database issue):D590-6.

22. Callahan BJ, McMurdie PJ, and Holmes SP. Exact sequence variants should replace operational taxonomic units in marker-gene data analysis. *Isme j.* 2017;11(12):2639-43.

23. Del Valle-Pinero AY, Van Deventer HE, Fourie NH, Martino AC, Patel NS, Remaley AT, et al. Gastrointestinal permeability in patients with irritable bowel syndrome assessed using a four probe permeability solution. *Clin Chim Acta.* 2013;418:97-101.
