# Supplementary figures and images for: Gut microbial diversity and functional characterization in people with alcohol use disorder: A case-control study
Source: PLoS One. 2024 Jun 12;19(6):e0302195. doi: 10.1371/journal.pone.0302195 (PMC11168635; doi:10.1371/journal.pone.0302195)

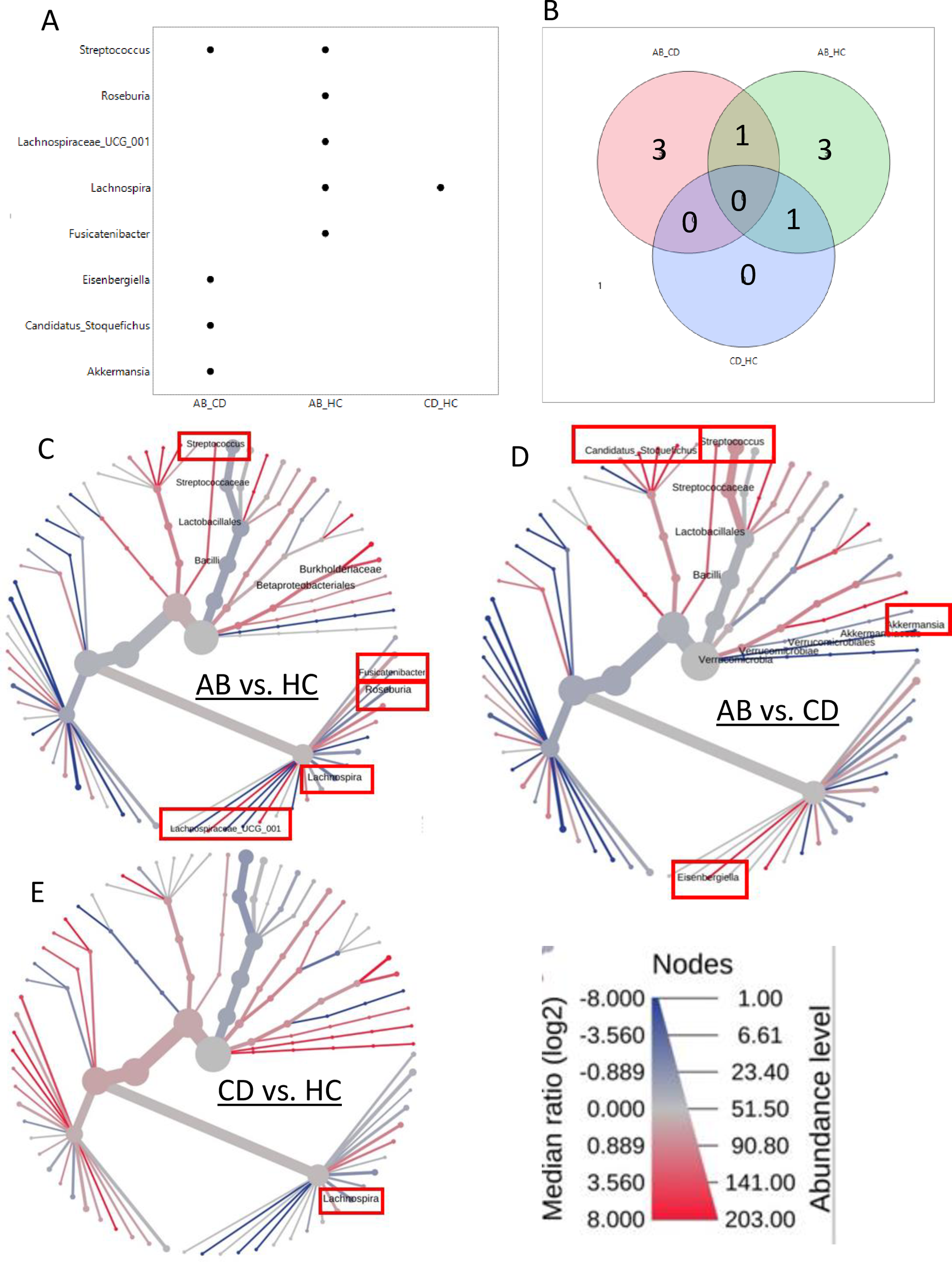

Supplement: S1 Fig — Pairwise comparison at the genus level between each group using Wilcoxon Test. (A) Dot plot of 9 genera (y-axis) found to be significant (p < .05, unadjusted) in pairwise comparison (x-axis). (B) Venn Diagram showing genera found to be significant that overlap between 3 pairwise tests. (C-E) The heat tree analysis leverages the hierarchical structure of taxonomic classifications to quantitatively (using the mean abundance) and statistically (using the non-parametric Wilcoxon Rank Sum test with p < .05) depict taxonomic differences between microbial communities. Red boxes show the end of the leaf (genus) in the heat tree for each plot. Taxa colored in red are more abundant in the AB (C, D) and CD (E) groups, taxa colored in blue are more abundant in the HC (C, E) and CD (D) groups. AB: Abstinent, CD: current drinkers, HC: healthy controls. (TIF) [file pone.0302195.s004.tif]

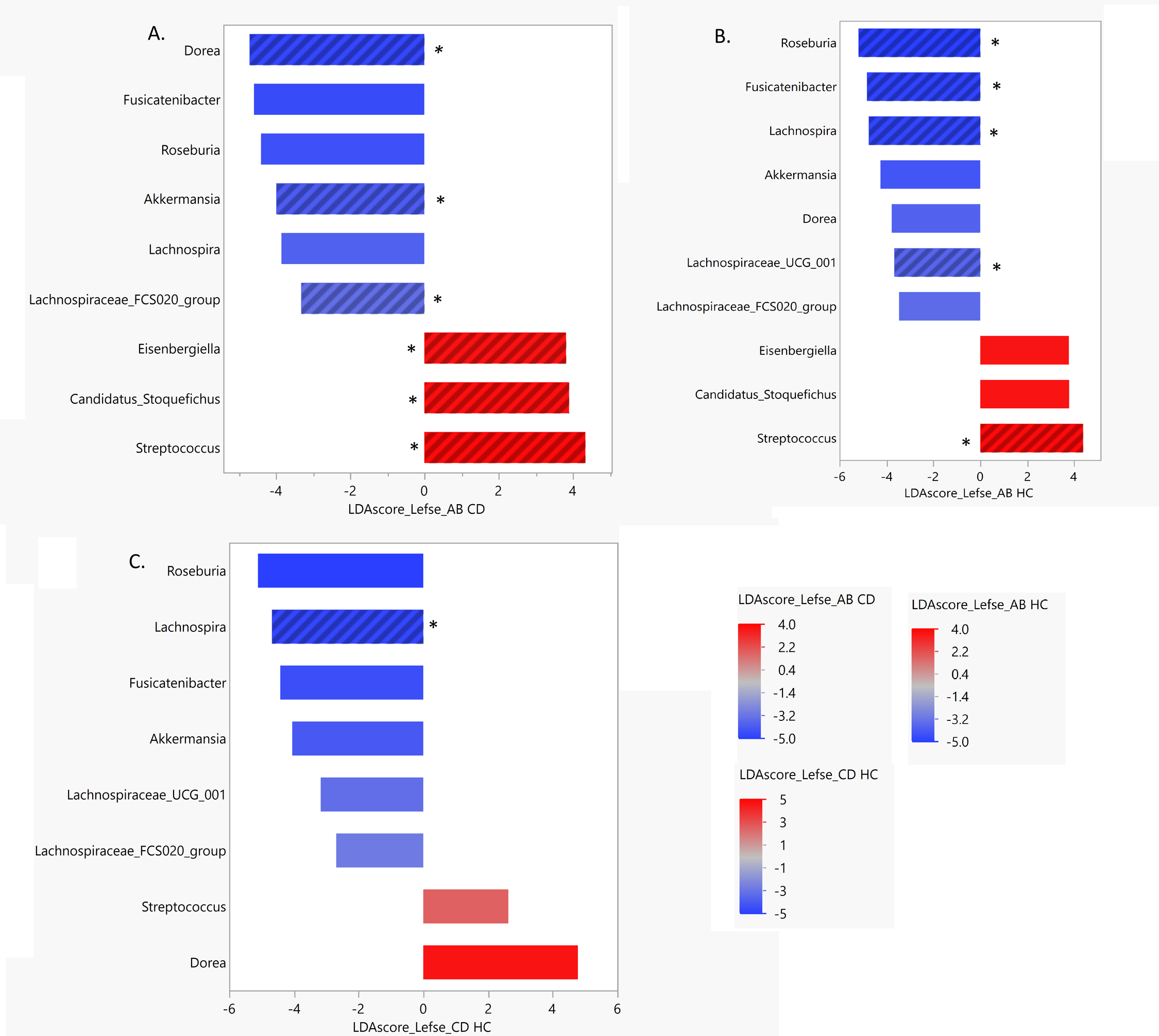

Supplement: S2 Fig — LEfSe LDA bar plots at the genus level of fecal microbial samples displaying the pairwise LDA scores between the three groups; abstinent group (AB), continuous alcohol drinking group (CD), and the healthy control group (HC). The bars represent the effect size (LDA) for a particular genus in a certain group. The length of the bar represents a log10 transformed LDA score. The heat maps show the LDA effect size from blue (negative) to red (positive). Shaded bars with an ‘*’ are genera that were found to be significant in the pairwise test (p < .05). (A) represents the pairwise comparison of AB to CD; (B) represents the pairwise comparison of AB to HC; and (C) represents the pairwise comparison of CD to HC. AB: Abstinent, CD: current drinkers, HC: healthy controls. (TIF) [file pone.0302195.s005.tif]

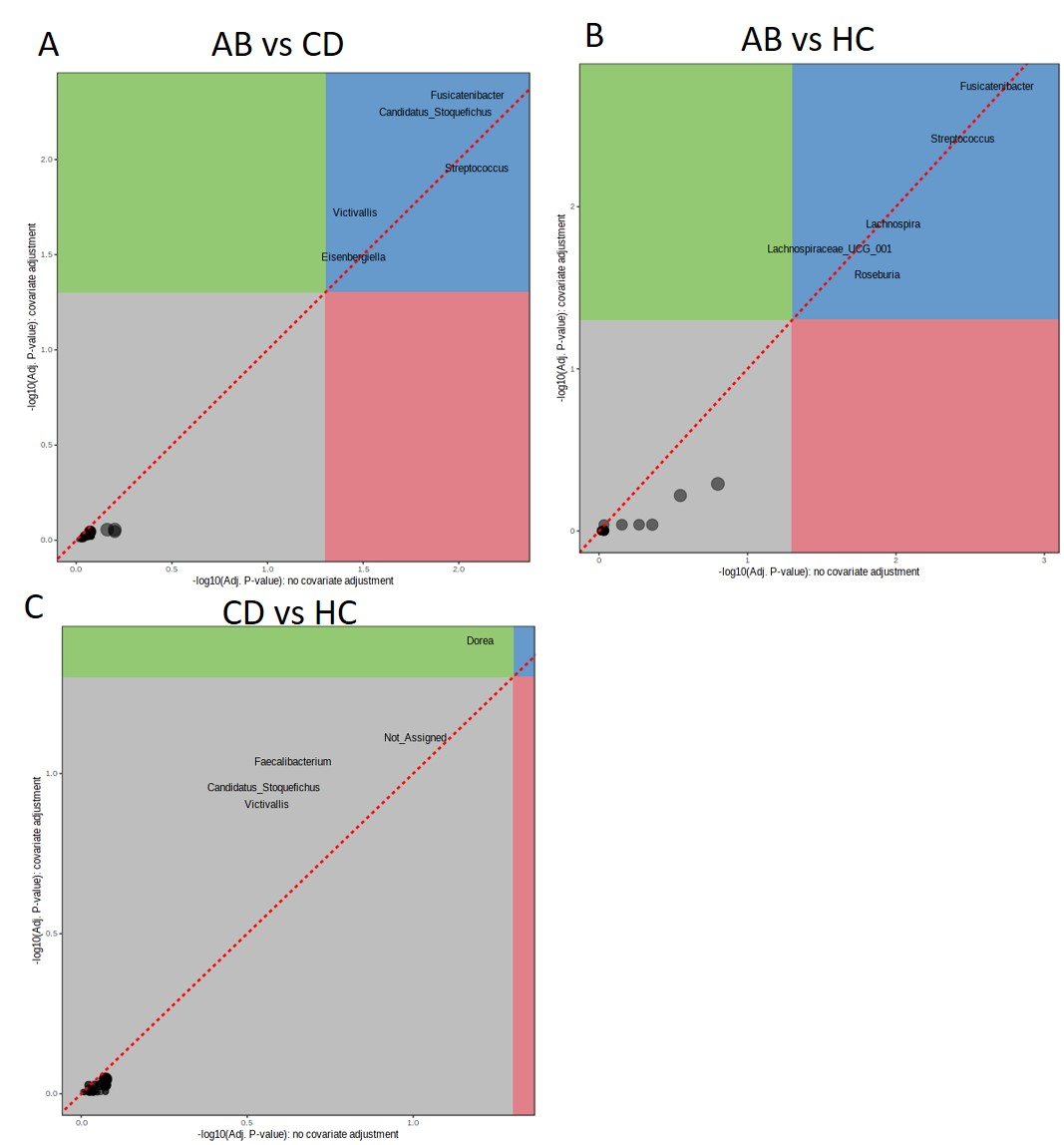

Supplement: S3 Fig — Results of covariates adjustment (y axis) and without adjustment (x axis). P values for the comparisons between (A) AB and CD, (B) AB and HC, and (C) CD and HC groups did not improve with covariate adjustment (BMI and age), resulting in no significantly different taxa. AB: Abstinent, CD: current drinkers, HC: healthy controls. (TIF) [file pone.0302195.s006.tif]

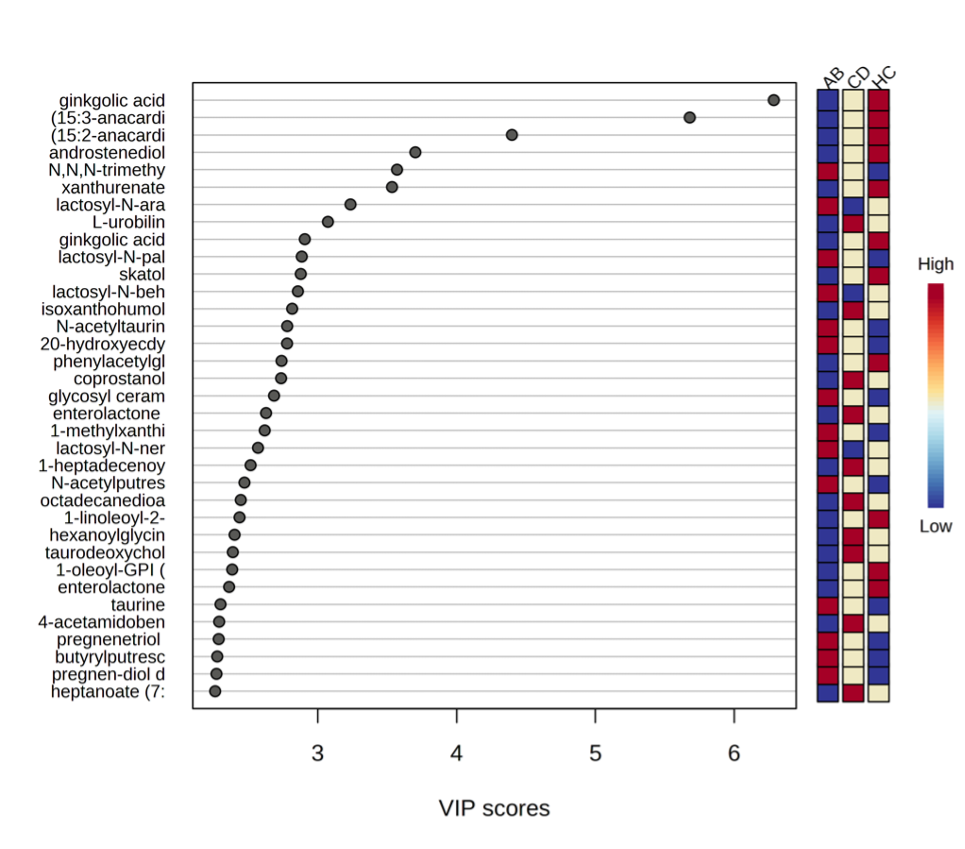

Supplement: S4 Fig — Variable importance in prediction (VIP) scores extracted by PLS-DA of metabolites and heatmap of the average scaled expression values of the indicated VIP metabolites in all samples. The top 30 metabolites driving differences between group are represented (lowest VIP score = 2.261). Metabolites in AB group samples show the lowest (in most cases) or the highest abundance compared to HC and CD groups. AB: Abstinent, CD: current drinkers, HC: healthy controls. (TIF) [file pone.0302195.s007.tif]

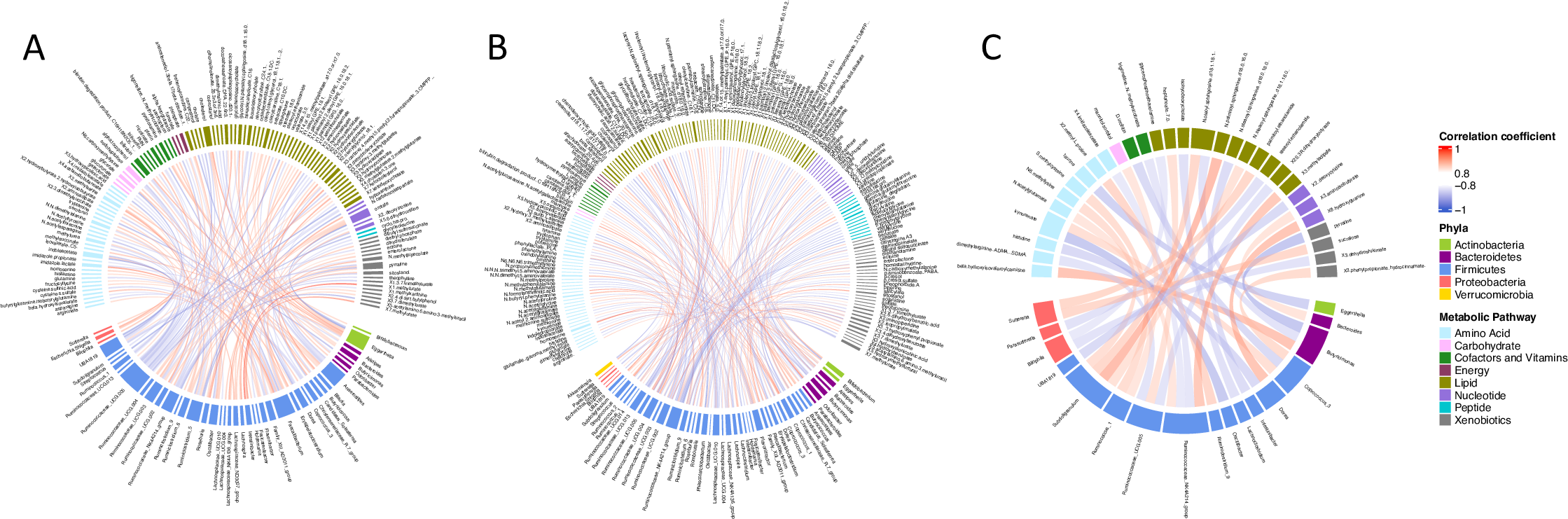

Supplement: S5 Fig — The correlations were computed within each group and a total of 149 significant correlation pairs were found within the ABs (A), 258 were found within the CDs (B), and 33 within the HCs (C). Most of the taxa within the AB group found to significantly correlate with metabolites, were represented in the CDs as well. Specifically, across the three comparisons, a high number of correlations were found in members from Lachnospiraceae and Oscillospiraceae families (Bacillota phylum): 62% in the CD group, 47% in the AB group, and 66% in the HC group. Streptococcus genus was found to be significantly more abundant in the AB group when compared to CD and HC groups. In the CD group Streptococcus genus was positively correlated with glyerolphosphoethanolamine (r = 0.950, p < .001), glycerophosphoserine (r = 0.917, p < .001), mannose (r = 0.917, p < .001), deoxycarnitine (r = 0.867, p = .002), and N,1, acetylspermidine (r = 0.850, p = .004). Conversely, in the same group we found negative correlations of Streptococcus with heptenedioate (C7:1-DC) (r = -0.865, p = .003), 2’-O-methylcytidine (r = -0.865, p = .003), and hexadecanedioate, C16 (r = -0.850, p = .004). In the AB group, Streptococcus genus correlated negatively with 7-ketocholesterol (r = -0.952, p < .001). Lachnospira genus, significantly less abundant in the AB group, was positively correlated with 3-hydroxyoctanoate (r = 0.931, p < .001) and with 1-palmitoyl-2-oleoyl-GPE (16:0/18:1) (r = 0.855, p = .002) in the AB group, while in the CD group correlated negatively with N-formylanthranilic acid (r = -0.900, p < .001), 2,hydroxybehenate (r = -0.900, p < .001), N-acetylglycine (r = -0.883, p = .002), urate (r = -0.867, p = .002), and pseudouridine (r = -0.850, p = .004). Lachnospira genus was positively correlated with indolepropionate (r = 0.867, p = .002) among the CDs. Roseburia genus, significantly less abundant in the AB group, correlated positively in the AB group with N,N,dimethylalanine (r = 0.867, p = .001), 1 [file pone.0302195.s008.tif]

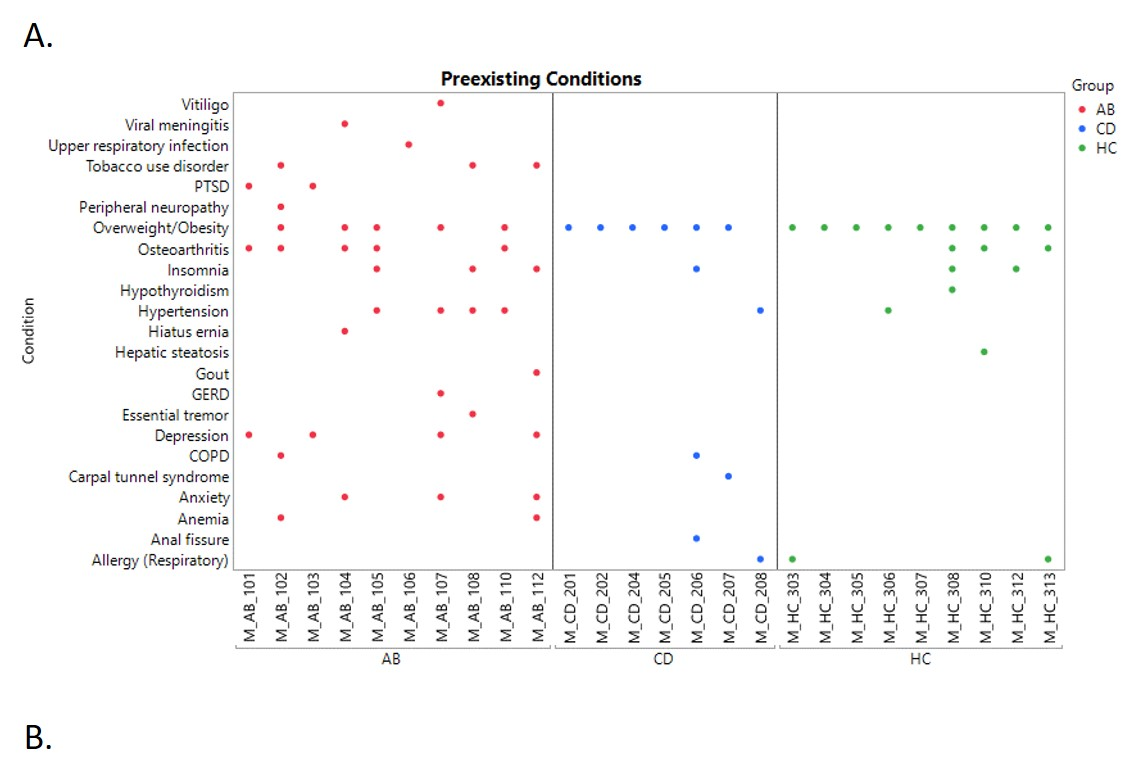

Supplement: S6 Fig — A) Reported participant preexisting conditions use over entire patient population. Blue dots indicate condition of AB participants, red dots indicate condition of CD participants and green dots indicate condition of HC participants. B) Preexisting conditions that overlap with Jackson et al. (2018) when selecting at a 20% FDR in S5 Table (Jackson et al., 2018). (AB: Abstinent, CD: current drinkers, HC: healthy controls). (TIF) [file pone.0302195.s009.tif]

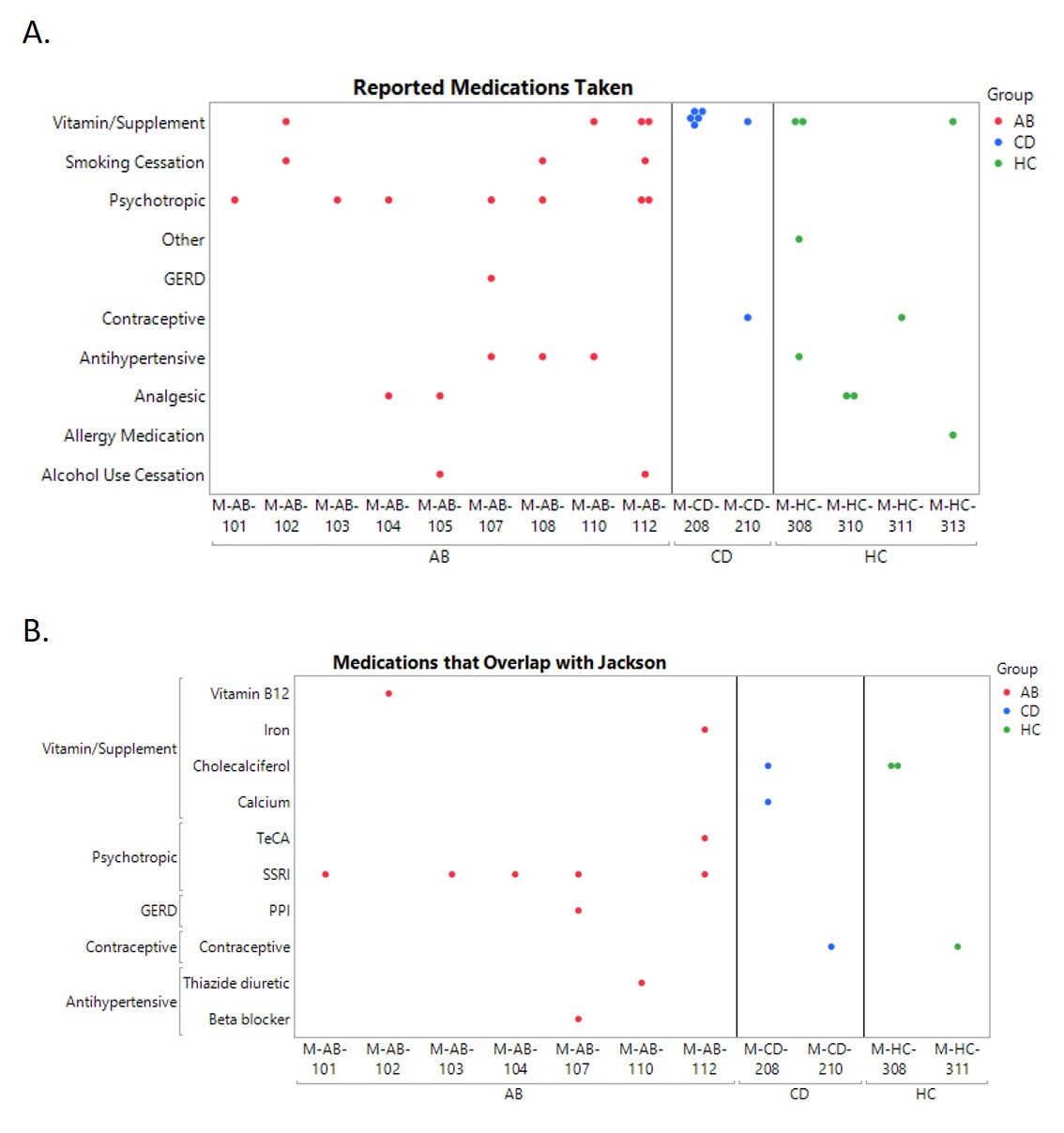

Supplement: S7 Fig — A) Reported participant medication use over entire patient population. Blue dots indicate medication taken by AB participants, red dots indicate medication taken by CD participants and green dot indicates medication taken by HC participants. B) Medications that overlap with Jackson et al. (2018) when selecting at a 20% FDR in S4 Table (AB: Abstinent, CD: current drinkers, HC: healthy controls). (TIF) [file pone.0302195.s010.tif]

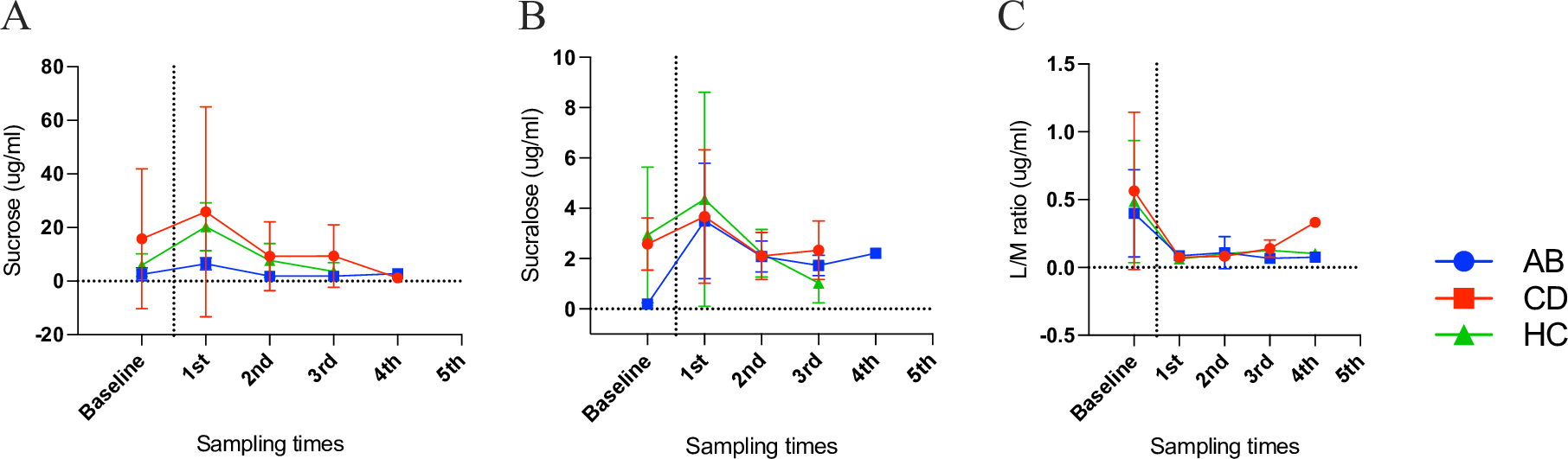

Supplement: S8 Fig — Detection of sucrose (A), Sucralose (B) and Lactulose/Mannitol ratio (C) by ultra-performance liquid chromatography mass spectrometry (UPLC-MS) analysis in suspected non-fasters participants. L/M ratio corresponded to the fractional excretion (FE) of lactulose and mannitol = (urine concentration from MS x total urine volume excreted)/sugar input). L/M ratio was calculated as FE lactulose/FE mannitol. Data are presented as mean values +/- SD. AB: Abstinent, CD: current drinkers, HC: healthy controls. Baseline = 40 minutes before starting the experiment, sampling times during the experiment: 1st ≈ 90 min, 2nd ≈ 100 min, 3rd ≈ 180 min, 4th ≈ 240 min, 5th ≈ 280 min AB: Abstinent, CD: current drinkers, HC: healthy controls. (TIF) [file pone.0302195.s011.tif]
